# Increasing BIN1 or decreasing DNM2 worsens pathology in a mouse model of dysferlinopathy

**DOI:** 10.1242/dmm.052918

**Published:** 2026-10-26

**Authors:** Alice Moncheaux, Emma Lafabrie, Patrice Goetz-Reiner, Maud Beuvin, Teresinha Evangelista, Jocelyn Laporte

**Affiliations:** ^1^ Institute of Genetics and Molecular and Cellular Biology (IGBMC), INSERM U1258, CNRS UMR7104, University of Strasbourg, 67404 Illkirch, France; ^2^ Institut Clinique de la Souris, PHENOMIN-ICS, 67404 Illkirch, France; ^3^ Neuromuscular Morphology Unit, Myology Institute, Groupe Hospitalier Universitaire Pitié-Salpêtrière, 75013 Paris, France; ^4^ Unité fonctionnelle de pathologie neuromusculaire, Department of Neuropathology, Sorbonne University, Pitié-Salpêtrière Hospital, Assistance Publique des Hôpitaux de Paris, 75013 Paris, France

**Keywords:** Myopathy, Dysferlin, Amphiphysin, Dynamin, Therapy

## Abstract

Dysferlinopathies are rare muscular dystrophies caused by loss-of-function variants in *DYSF*, which encodes dysferlin, leading to progressive muscle weakness. Although dysferlin is known for its role in membrane repair, emerging evidence suggests additional functions in membrane remodeling and T-tubule organization. Therapeutic strategies targeting the BIN1–DNM2 axis have successfully rescued T-tubule and force defects in centronuclear myopathies, raising the possibility of broader applicability. Here, we evaluated whether BIN1 overexpression or DNM2 downregulation could improve disease features in dysferlin-deficient mice through genetic crosses. Unexpectedly, both modulations worsened the *Dysf^−/−^* phenotype. BIN1 overexpression increased necrosis, fibrosis and circulating MYOM3 levels. DNM2 downregulation produced an even more severe phenotype, including additional reduced force and fiber calcification. Importantly, DNM2 protein levels were intrinsically elevated in *Dysf^−/−^* mice and biopsies from patients with dysferlinopathy. Because both strategies reduced DNM2 levels and worsened the phenotype, the elevated DNM2 levels likely represent a compensatory rather than pathogenic mechanism. Our findings demonstrate that therapeutic modulation of the BIN1–DNM2 axis has disease-specific consequences. In dysferlinopathy, DNM2 upregulation may represent a compensatory mechanism required for membrane stability, underscoring the need for context-dependent therapeutic strategies.

## Teaser

**Summary:** Using a *Dysf*^−/−^ mouse model of dysferlinopathy, this study provides mechanistic insight into how therapeutic strategies effective in one muscle disease can be deleterious in another, highlighting the importance of disease-specific interventions.

## INTRODUCTION

Dysferlinopathies are a group of rare autosomal recessive muscular dystrophies caused by loss-of-function variants in the *DYSF* gene, which encodes dysferlin. The clinical spectrum includes several overlapping phenotypes such as Miyoshi distal myopathy [Online Mendelian Inheritance in Man (OMIM) #254130], limb-girdle muscular dystrophy R2 dysferlin-related (LGMDR2; formerly LGMD2B; OMIM #253601), distal myopathy with anterior tibial onset (OMIM #606768), and isolated or minimally symptomatic hyperCKemia (Cheema et al., 2025). Disease onset typically occurs in late adolescence or early adulthood and is characterized by progressive muscle weakness and wasting, most commonly affecting either distal posterior leg muscles or proximal limb-girdle muscles. Patients frequently present with marked hyperCKemia and exercise intolerance, and may show inflammatory changes on muscle biopsy. Over time, progressive degeneration of muscle fibers leads to fibrofatty replacement of muscle tissue, a process that can be monitored using muscle magnetic resonance imaging (Harris et al., 2016; Bolano-Diaz et al., 2025; Anwar and Yokota, 2024). Currently, no disease-modifying therapy is available for dysferlinopathy.

Dysferlin is a transmembrane protein highly expressed in striated muscle, where it contributes to calcium-activated vesicle-mediated membrane repair in cooperation with annexins and mitsugumin 53 (MG53; also known as TRIM72) (Lennon et al., 2003; Cai et al., 2009; Bloch et al., 2025). Like other Ferlin family members, dysferlin possesses several C2 domains, including a C2A domain that binds MG53, AHNAK and α-tubulin, as well as a calpain-cleavable region composed of the C2F and C2G domains, which is separated from the rest of the protein upon membrane injury and is referred to as mini-dysferlin.

Dysferlin loss has been described as primarily affecting membrane repair, leading to the dystrophic phenotype. Therefore, therapeutic strategies have largely focused on restoring membrane repair capacity, either indirectly or through reconstitution of dysferlin expression. However, *DYSF* is a large gene with 6.9 kb mRNA (Liu et al., 1998) that exceeds the packaging capacity of a single adeno-associated virus (AAV). Dual AAV-mediated *Dysf* transfer was shown to partially improve the phenotype in a dysferlin-deficient mouse model, but full dysferlin production remained challenging, with only up to 4% of total protein produced (Lostal et al., 2010). This observation drove the development of mini-dysferlin gene therapy approaches. Although mini-dysferlin or other nano-dysferlin expression successfully restored membrane repair and sarcolemmal integrity *in vitro*, it failed to significantly rescue the phenotype of dysferlin-deficient mice, i.e. dystrophy and muscle weakness, or calcium signaling *in vitro*, indicating that dysferlinopathy involves a more complex pathomechanism than impaired membrane repair alone (Lostal et al., 2012; Muriel et al., 2024).

A previous study has shown that dysferlin loss leads to T-tubules defects in a mouse model of dysferlinopathy (Klinge et al., 2010a). T-tubules are specialized invaginations of the sarcolemma in muscle fibers that ensure the rapid propagation of action potentials and are essential for excitation–contraction (E-C) coupling. T-tubules defects have already been described as causing muscle weakness by disrupting E-C coupling (Al-Qusairi et al., 2009). Dysferlin was implicated in T-tubule biogenesis, along amphiphysin 2 (BIN1), dynamin 2 (DNM2) and caveolin-3 (Cav-3), and was shown to tubulate membranes *in vitro* similarly to BIN1 (Hofhuis et al., 2017; Lemerle et al., 2023; Al-Qusairi and Laporte, 2011; Ji et al., 2025; Cowling et al., 2017). Variants in the encoding genes for either of these proteins cause muscle diseases, namely centronuclear myopathy (CNM) for *BIN1* loss of function and *DNM2* gain of function, and caveolinopathy for *CAV3* loss of function (Minetti et al., 1998; Gómez-Oca et al., 2021; Nicot et al., 2007; Bitoun et al., 2005). Notably, BIN1 has been shown to inhibit DNM2 activity *in vitro* and *in vivo* (Cowling et al., 2017; Goret et al., 2025). Accordingly, overexpressing BIN1 or downregulating DNM2 similarly restores proper T-tubule structure and muscle force in mouse models of CNM (Ji et al., 2025; Li et al., 2022; Cowling et al., 2014; Silva-Rojas et al., 2022; Lionello et al., 2019, 2022; Buono et al., 2018).

Therefore, we hypothesized that modulating BIN1 or DNM2 in a dysferlinopathy mouse model could improve T-tubule organization and the muscle phenotype. Given the rarity of dysferlinopathies, the possibility of a common therapeutic strategy applicable to multiple myopathies would be particularly beneficial for patients.

## RESULTS

### Characterization of *Dysf^−/−^* mice reveals no improvement in muscle force upon BIN1 increase

To evaluate whether increasing BIN1 expression ameliorates dysferlinopathy, we used the BLA/J mouse model (Lostal et al., 2010; Nagy et al., 2017), which carries an ETn retrotransposon insertion in the fourth intron of the *Dysf* gene, resulting in complete loss of dysferlin expression. Mice homozygous for this insertion are hereafter referred to as *Dysf*^−/−^. We assessed *in vivo* and histological phenotypes at 6 months of age, given that muscle defects were previously reported to begin around 4 months (Nagy et al., 2017). Similarly to patients with LGMDR2, *Dysf^−/−^* mice displayed predominantly proximal muscle involvement, with the psoas muscle being one of the most affected muscles (Lostal et al., 2010; Bouchard et al., 2023; Hornsey et al., 2013) (Fig. S1). Pathological features were also evident in other muscles such as the tibialis anterior (TA) muscle, which showed necrosis and myofibers with internalized nuclei, indicative of regenerating fibers (Fig. S1).

*Dysf^+/−^* mice were crossed with transgenic mice carrying a human *BIN1* transgene (*TgBIN1*) to obtain healthy [wild-type (WT)], diseased (*Dysf^−/−^*), treated (*Dysf^−/−^TgBIN1*) and control (*TgBIN1*) offspring. Total BIN1 mRNA and protein expression levels in the TA muscle were increased 4- to 6-fold in *Dysf^−/−^TgBIN1* and TgBIN1 mice compared to endogenous BIN1 levels in WT and *Dysf^−/−^* mice (Fig. 1A,B), consistent with previously published data on *TgBIN1* (Lionello et al., 2019).

**Fig. 1.**
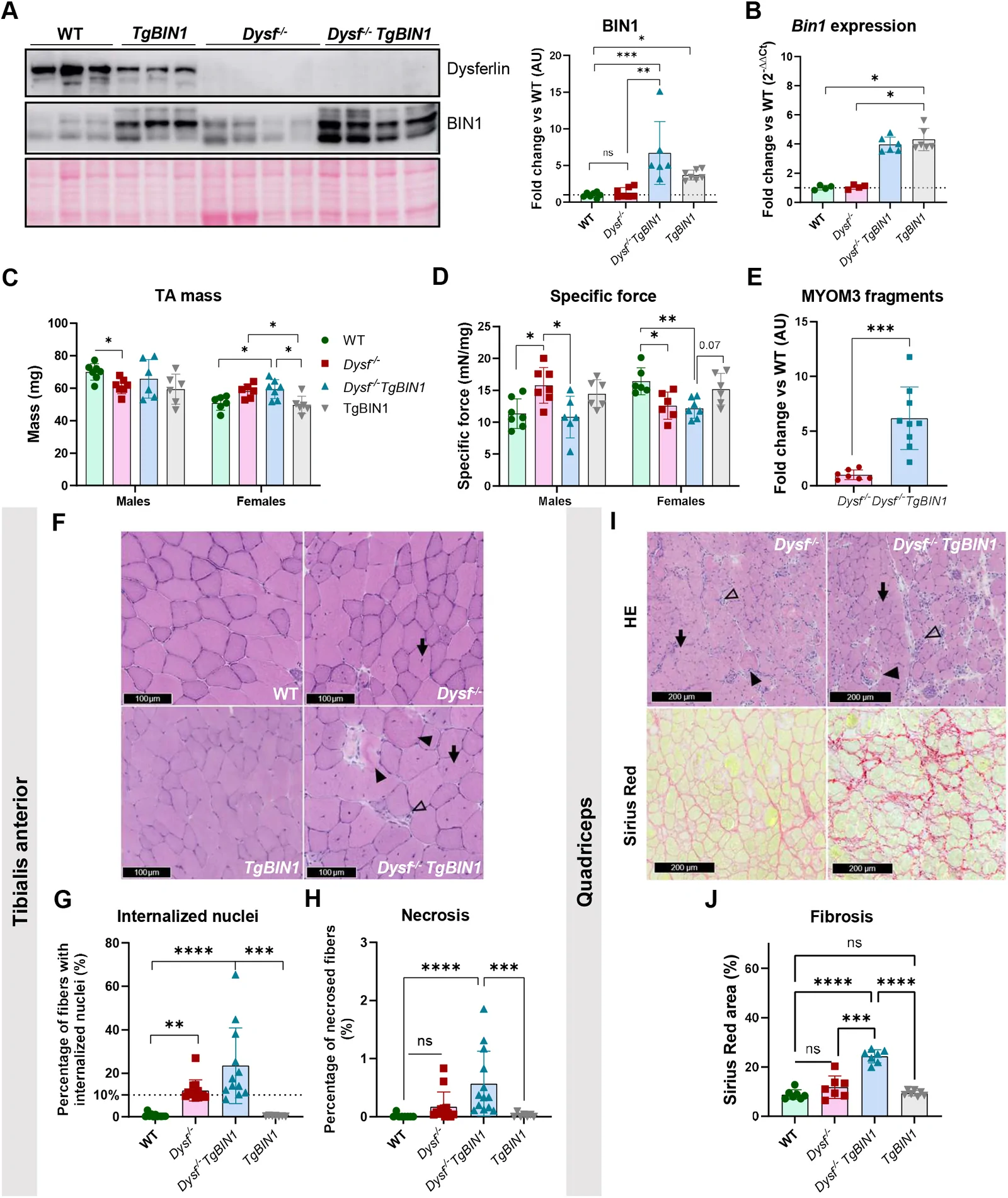
**BIN1 overexpression in *Dysf^−/−^* mice does not rescue the phenotype and partially worsens dystrophic features.** (A,B) Quantification of BIN1 protein (A) and mRNA (B) in tibialis anterior (TA) extracts at 6 months of age. (A) *n*=7 or 6 (KOTG). (B) *n*=4 (WT, KO) or 6. Kruskal–Wallis with multiple comparison test. (C) Mass of TAs used for *in situ* muscle force measurements. One-way ANOVA or Brown–Forsythe and Welch test. (D) *In situ* muscle force measurements at 6 months in the TA after sciatic nerve stimulation at 150 Hz. (C,D) *n*=6-7 /sex/genotype. One-way ANOVA with Tukey's multiple comparisons. (E) Quantification of MYOM3 fragments in the plasma by western blotting at 6 months of age. *n*=7 (KO) or 9 (KOTG). Unpaired *t*-test with Welch's correction. (F) Representative images of HE-stained transversal TA section at 6 months. (G,H) Quantification of the percentage of fibers with internalized nuclei (G) or undergoing necrosis (H). Kruskal–Wallis with Dunn's multiple *t*-test. (G) *n*=11 (WT), 13 (KO), 12 (KOTG), 7 (TG). (H) *n*=8 (WT), 13 (KO), 13 (KOTG), 7 (TG). (I) Representative images of HE- or Sirius Red-stained transversal quadriceps section at 6 months. (J) Fibrosis is measured as the area occupied by Sirius Red staining. *n*=7. Brown–Forsythe and Welch test with Dunnett's multiple comparisons. ns, not significant; *P<0.05; **P<0.01; ***P<0.001; ****P<0.0001. Each dot represents a mouse. In F and I, black arrows indicate fibers with at least one internalized nucleus, black arrowheads indicate necrosis, and empty arrowheads indicate immune cell infiltration. AU, arbitrary units; HE, Hematoxylin–Eosin; KO, *Dysf^−/−^*; KOTG, *Dysf^−/−^TgBIN1*; TG, *TgBIN1.* Force measurements and histological analyses were performed once.

Intrinsic muscle force was assessed in all genotypes and both sexes by *in situ* stimulation of the sciatic nerve and measurement of the resulting TA force. At 6 months of age, *Dysf^−/−^* males, but not females, showed a significant reduction in TA muscle mass (Fig. 1C). They also produced greater specific muscle force, with a 38% increase compared to that in WT males (Fig. 1D). The increased strength we observed might reflect an early phase of the disease, similar to reports that some patients show unusually high physical capabilities before symptom onset (Harris et al., 2016; Klinge et al., 2010b). *Dysf^−/−^TgBIN1* males, however, had decreased strength compared to that of *Dysf^−/−^* males (31% reduction) and no significant difference from that of controls.

In contrast, female mice exhibited a distinct phenotype at this age. TA muscle mass was increased in *Dysf^−/−^* and *Dysf^−/−^TgBIN1* mice relative to that in at least one control group (Fig. 1C), and both *Dysf^−/−^* and *Dysf^−/−^TgBIN1* females exhibited a 20-23% reduction in specific maximal force compared to that in their respective control, showing that BIN1 overexpression did not rescue muscle weakness (Fig. 1D; Fig. S2A).

As *in situ* force measurements at 6 months of age may reflect an early stage of the disease progression in distal muscles, we assessed TA muscle force at 1 year in a limited number of animals. At 1 year, the phenotypes of males and females appeared similar, and absolute force in *Dysf^−/−^* and *Dysf^−/−^TgBIN1* animals was lower than that in WT or *TgBIN1* animals, respectively, although the differences were not significant*.* When normalized to the TA mass, the specific force of *Dysf^−/−^* and *Dysf^−/−^TgBIN1* mice was significantly decreased compared to that in at least one control group (Fig. S2B,C).

Overall, *Dysf^−/−^* and *Dysf^−/−^TgBIN1* force measurements were similarly decreased compared to those in controls at 1 year of age, as well as in females at 6 months, indicating that BIN1 overexpression could not rescue the muscle weakness associated with dysferlin loss.

### Increasing BIN1 in *Dysf^−/−^* mice exacerbates some dystrophic features of mice with dysferlinopathy

To further characterize the effect of increasing BIN1 in dysferlinopathy, we quantified MYOM3 fragments in the plasma, which represent a circulating marker for dystrophies (Rouillon et al., 2015). In the control condition (WT and *TgBIN1*), MYOM3 fragments were nearly undetectable; thus, only the pathologic genotypes were quantitatively compared (Fig. S2D). *Dysf^−/−^* mice displayed a clear increase in circulating MYOM3 fragments compared to those in WT mice (Fig. S2D). BIN1 overexpression in *Dysf^−/−^TgBIN1* mice caused a 5-fold increase in MYOM3 fragments (Fig. 1E). These data suggest that dystrophic features are not corrected following BIN1 increase, and this was further supported by histological analysis. Proper nuclei localization was not restored by BIN1 modulation in the *Dysf^−/−^TgBIN1* mice (Fig. 1F,G), and we even observed an increase in necrotic fibers in the TA of *Dysf^−/−^TgBIN1* mice compared to those in WT mice (Fig. 1F,H), as well as increased fibrosis in the quadriceps compared to that in *Dysf^−/−^* mice (Fig. 1I,J). Psoas histology was also not rescued by increasing BIN1, and we observed pronounced fiber diameter heterogeneity on top of the previously described hallmarks of dystrophy in *Dysf^−/−^TgBIN1* mice (Fig. S2E).

Overall, BIN1 overexpression did not improve proximal or distal histological features in *Dysf*^−/−^ mice, and led to a more pronounced phenotype, with increased fibrosis and dystrophic biomarker MYOM3.

### DNM2 protein levels are associated with disease severity in dysferlinopathy

To better understand how increasing BIN1 would accentuate the phenotype of *Dysf*^−/−^ mice, we looked at other proteins functionally related to BIN1 and dysferlin, such as DNM2 and Cav-3.

DNM2 protein levels showed a non-significant trend toward elevation in the TA of *Dysf*^−/−^ mice, with most animals displaying a ∼2-fold increase (Fig. 2A). Cav-3 protein levels were similar in *Dysf*^−/−^ and WT TA muscles (Fig. 2A). Increasing BIN1 in *Dysf^−/−^TgBIN1* mice led to a strong increase in DNM2 and Cav-3 levels compared to those in other groups, including *Dysf*^−/−^ mice (Fig. 2A). This increase at the protein level was not linked to an increase at the RNA level, suggesting that it was not driven by a compensatory transcriptional mechanism (Fig. 2B,C), but could instead result from post-transcriptional dysregulations or altered protein stability.

**Fig. 2.**
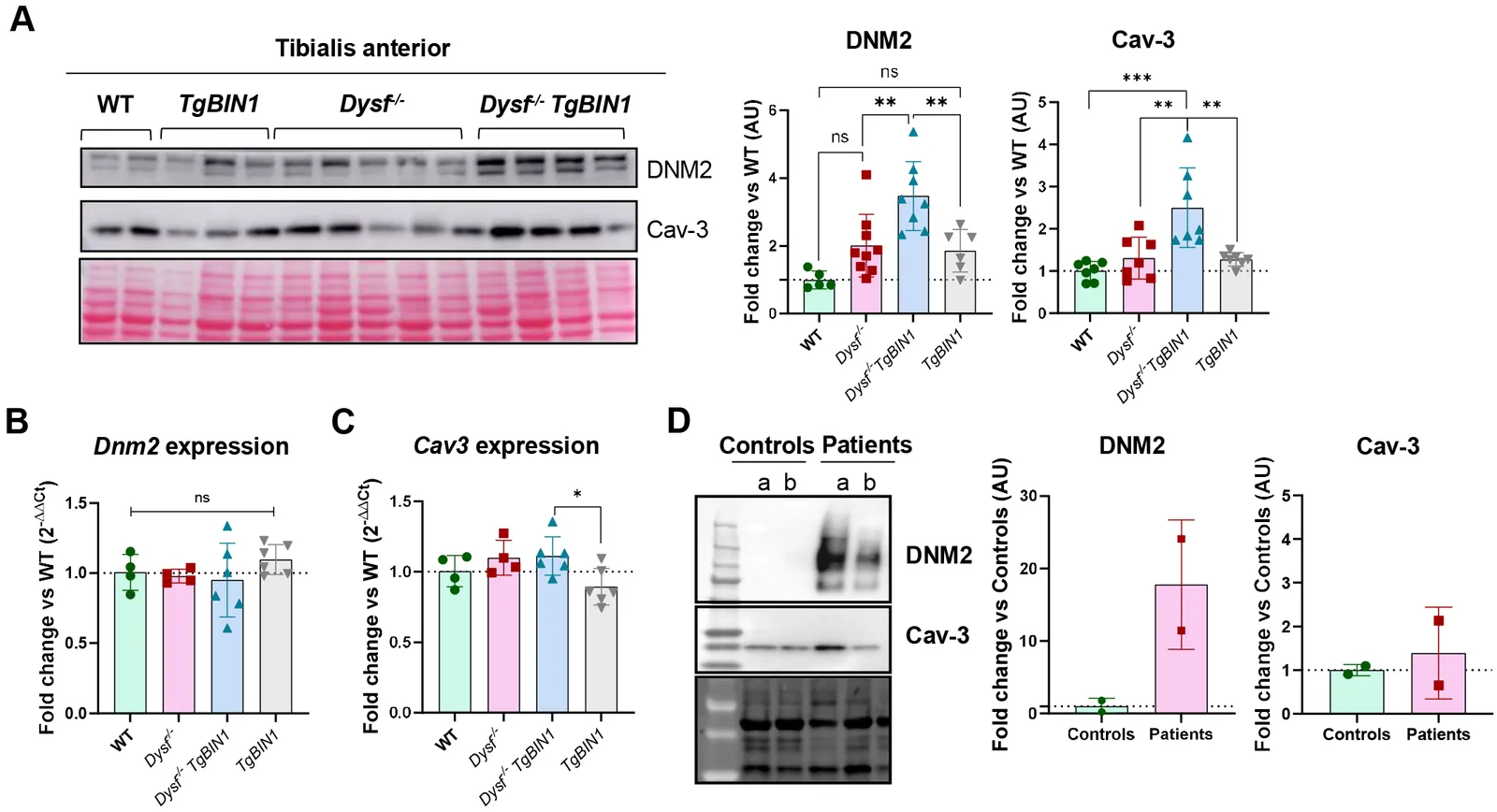
**DNM2 protein level is associated with disease severity in dysferlinopathy.** (A-C) Representative western blot images (A) and quantification of DNM2 and Cav-3 protein (A) and mRNA (B,C) levels in TA extracts at 6 months of age, representative of three independent experiments. One-way ANOVA with Tukey's test (A,C). Brown–Forsythe ANOVA (B). (A, left) *n*=5 (WT), 6 (KO), 9 (KOTG), 8 (TG). (A, right) *n*=7. (B,C) *n*=4 (WT), 4 (KO), 6 (KOTG), 6 (TG). ns, not significant; *P<0.05; **P<0.01; ***P<0.001. Each dot represents a mouse. (D) Representative western blot images (left) and quantification of DNM2 (middle) and Cav-3 (right) protein levels in patient and control biopsies (a, 20 years old; b, 55 years old). Each dot represents an individual. KO, *Dysf^−/−^*; KOTG, *Dysf^−/−^TgBIN1*; TG, *TgBIN1*.

To assess whether BIN1 overexpression had the same molecular impact in a proximal muscle, we quantified DNM2 and Cav-3 in the psoas muscle. In this severely affected muscle, DNM2, but not Cav-3, protein levels were significantly elevated in *Dysf*^−/−^ mice compared with those in WT controls (Fig. S3A,B). Similar dysregulation of DNM2 and Cav-3 was observed in the psoas of *Dysf^−/−^TgBIN1* mice, mirroring the alterations detected in the distal TA muscle. To note, DNM2 localization in WT and *Dysf^−/−^* muscles did not differ (Fig. S4).

As DNM2 elevation appeared as a hallmark of disease severity in the mouse model, we investigated its levels in muscle biopsies from patients with dysferlinopathy. Patient biopsies confirmed marked increase in DNM2 protein in the absence of dysferlin (11- and 24-fold in the two analyzed patients), suggesting a conserved impact of DNM2 on the pathomechanism of dysferlinopathies (Fig. 2D). Cav-3 levels, however, were not different between patient and control biopsies.

Together, these results indicate that DNM2 is markedly upregulated in the areas most severely affected in dysferlinopathy (proximal muscle or following BIN1 overexpression), whereas Cav-3 upregulation appears to be specific to the combined modulation of BIN1 and dysferlin.

### DNM2 downregulation exacerbates muscle weakness and induces calcification in *Dysf^+/−^* mice

As DNM2 increase is described to be deleterious in skeletal muscle (Cowling et al., 2011), and its downregulation has proven efficient in rescuing four genetic forms of CNM (Li et al., 2022; Cowling et al., 2014; Silva-Rojas et al., 2022; Buono et al., 2018; de Carvalho Neves et al., 2023), we investigated whether DNM2 downregulation could improve the phenotype of *Dysf^−/−^* mice. To do so, we crossed *Dysf^+/−^* with mice expressing only half of DNM2, *Dnm2^+/^*^−^, previously validated to rescue CNM mouse models (Cowling et al., 2014). We confirmed that heterozygous *Dnm2* knockout was able to normalize DNM2 protein level in the TA of *Dysf^−/−^Dnm2^+/−^* mice (Fig. 3A,B).

**Fig. 3.**
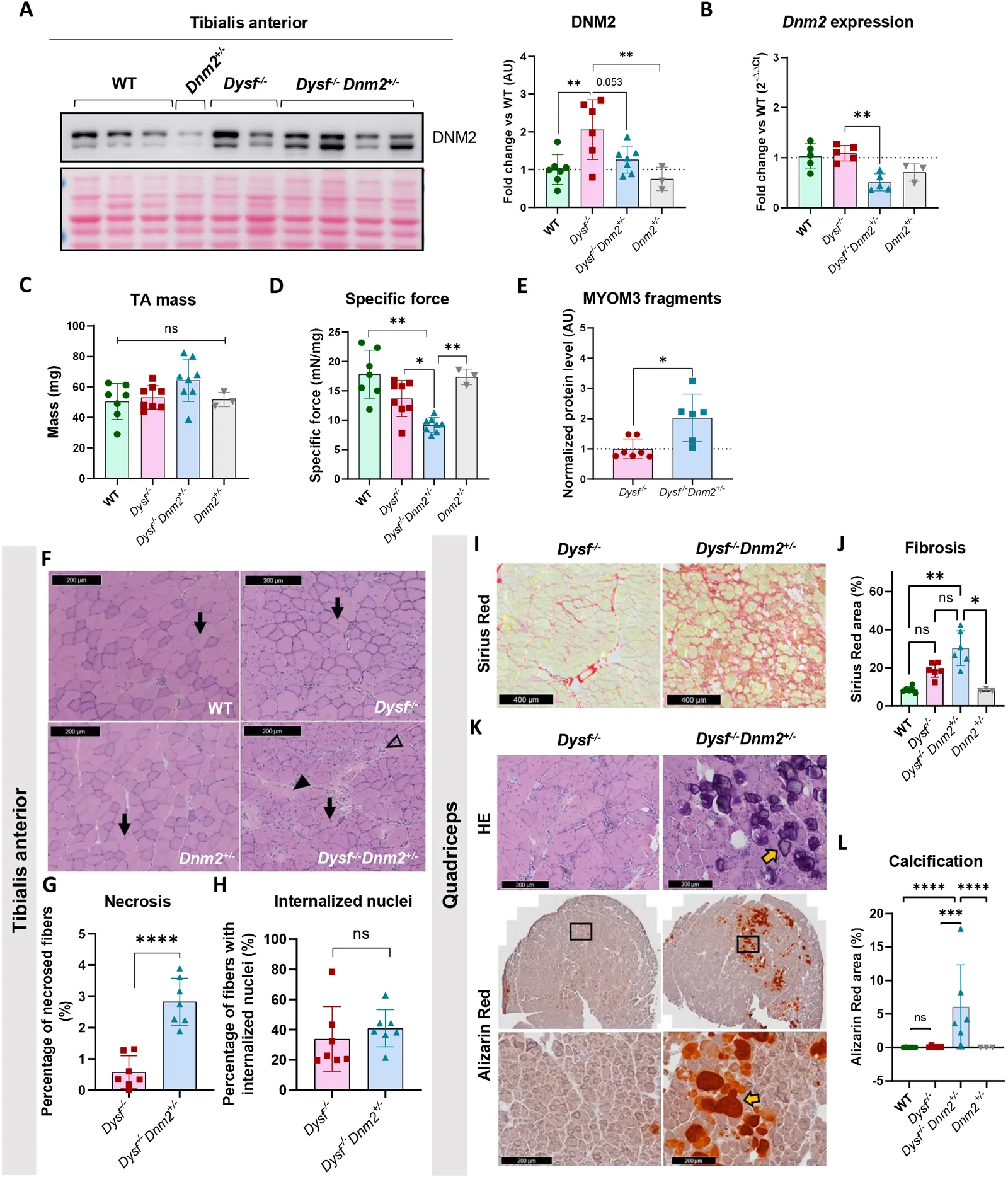
**DNM2 downregulation exacerbates most phenotypes of *Dysf^−/−^* mice.** (A,B) Quantification of DNM2 protein (A) and mRNA (B) levels in the TA at 1 year of age. Ordinary one-way ANOVA. (A) *n*=7 (WT; KOHET), 6 (KO), 3 (HET). (B) *n*=5 (WT; KO; KOHET), 3 (HET). (A) DNM2 detection was performed on the same membrane as the one used in Fig. 6C. (C) Mass of TAs used for *in situ* muscle force measurements. Ordinary one-way ANOVA. (D) *In situ* muscle force measurements in the TA after sciatic nerve stimulation at 150 Hz at 1 year of age. Brown–Forsythe and Welch test. (C,D) *n*=7 (WT), 8 (KO; KOHET), 3 (HET). (E) Quantification of MYOM3 fragments in the plasma by western blotting at 8 months of age. Mann–Whitney test. *n*=7 (KO), 6 (KOHET). (F) Representative images of HE-stained transversal TA section at 1 year. Black arrows indicate fibers with at least one internalized nucleus. Black arrowheads indicate necrosis. Empty arrowhead indicates immune cell infiltration. (G,H) Quantification of the percentage of fibers undergoing necrosis (G) or with internalized nuclei (H). *n*=7. Unpaired *t*-test (on lognormally transformed data for internalized nuclei). (I) Representative images of transversal quadriceps sections at 1 year stained with Sirius Red. (J) Fibrosis is measured as the area occupied by Sirius Red staining as a percentage of the total tissue area. Kruskal–Wallis with multiple comparison test. (K) Representative images of HE- and Alizarin Red-stained transversal quadriceps section at 1 year. Yellow arrows indicate fiber calcification. (L) Fiber calcification is measured as the area occupied by Alizarin Red staining as a percentage of the total tissue area. Ordinary one-way ANOVA with multiple comparison. (J,L) *n*=6 (WT; KO; KOHET), 3 (HET). ns, not significant; *P<0.05; **P<0.01; ***P<0.001; ****P<0.0001. Each dot represents a mouse. KO, *Dysf^−/−^*; KOHET, *Dysf^−/−^Dnm2^+/−^*; HET, *Dnm2^+/−^.* Force measurements and histological analyses were performed once.

As TA muscle weakness was stronger at 1 year of age in *Dysf*^−/−^ mice than that at 6 months, *Dysf^−/−^Dnm2^+/−^* mice underwent *in situ* muscle force measurements in the TA at 1 year. Statistical analysis allowed male and female data to be pooled, thus reducing the number of animals needed (Fig. S5A,B). TA mass did not differ across genotypes (Fig. 3C). *Dysf^−/−^Dnm2^+/−^* mice exhibited reduced force compared to WT and *Dnm2^+/−^* controls, with or without normalization to TA mass (47% or 34% reduction compared to that in *Dnm2^+/−^* mice, respectively) (Fig. 3D; Fig. S5C). Additionally, DNM2 downregulation significantly decreased specific force in *Dysf^−/−^* mice by 32% compared to that in diseased *Dysf*^−/−^ mice (Fig. 3D).

This worsening in muscle weakness was accompanied by elevated MYOM3 levels in the plasma in *Dysf^−/−^Dnm2^+/−^* mice compared to that in *Dysf^−/−^* mice (Fig. 3E). Histological analyses of TA muscles revealed an increase in necrotic fibers in *Dysf^−/−^Dnm2^+/−^* mice compared to those in *Dysf^−/−^* mice, without any impact on nuclei position (Fig. 3F-H). Investigation of quadriceps following Sirius Red staining showed an increase in fibrosis upon DNM2 downregulation in the *Dysf^−/−^Dnm2^+/−^* mice compared to that in the controls, with some mice exhibiting up to 40% of fibrotic area, outside the *Dysf^−/−^* range of ∼20% (Fig. 3I,J). Hematoxylin–Eosin (HE) and Alizarin Red staining in the quadriceps and psoas muscles led to the discovery of a new histological phenotype in the *Dysf^−/−^Dnm2^+/−^* mice compared to compared to *Dysf^−/−^* mice, attributed to fiber calcification (Fig. 3K,L; Fig. S5D). As the calcification in the quadriceps was a strong read-out of the *Dysf^−/−^Dnm2^+/−^* phenotype, we looked at the quadriceps of 1-year-old *Dysf^−/−^TgBIN1* mice. No calcification was detected upon HE staining, and few occurrences could be seen upon Alizarin Red staining, to a similar extent as in *Dysf^−/−^* mice (Fig. S6).

Overall, DNM2 downregulation significantly worsened muscle weakness and histological features in *Dysf^−/−^* mice (Table 1) and appeared to be more deleterious than BIN1 overexpression at 1 year of age.

**Table 1.** Effects of BIN1 and DNM2 modulations in the *Dysf^−/−^* mouse model of dysferlinopathy

Phenotypes	Modulation	
	5-fold BIN1 protein level increase	Half DNM2 protein level
TA weakness at 1 year of age	No rescue	Worsening ↓ force in *Dysf^−/−^Dnm2^+/−^* compared to all other groups
Nuclei internalization in the TA	No rescue	No rescue
Fiber necrosis in the TA	Partial worsening ↑ necrosis compared to controls but not to *Dysf^−/−^*	Worsening 6-fold ↑ in *Dysf^−/−^Dnm2^+/−^* compared to *Dysf^−/−^*
Fibrosis in the quadriceps	Worsening 2-fold ↑ in *Dysf^−/−^TgBIN1* compared to *Dysf^−/−^*	Partial worsening ↑ fibrosis compared to controls but not to *Dysf^−/−^*
MYOM3 circulating fragments	Worsening 5-fold ↑ in *Dysf^−/−^TgBIN1* compared to *Dysf^−/−^*	Worsening 2-fold ↑ in *Dysf^−/−^Dnm2^+/−^* compared to *Dysf^−/−^*
Calcification in the quadriceps at 1 year of age	No phenotype	Worsening ↑ calcification in *Dysf^−/−^Dnm2^+/−^* compared to all other groups

BIN1 modulation was performed at 6 months of age, unless otherwise specified in the ‘Phenotypes’ column, whereas DNM2 modulation was performed at 1 year of age, except for MYOM3 measures done at 8 months of age. TA, tibialis anterior.

### The dystrophic niche is not normalized by DNM2 downregulation

As we observed pronounced histological alterations, with fibrosis, calcification and increased inter-fiber nuclei, suggestive of immune cell infiltration, we examined muscle environment dysregulation in *Dysf^−/−^* and *Dysf^−/−^Dnm2^+/−^* quadriceps muscle. We assessed macrophage infiltration and distribution (immunofluorescence) alongside transcriptional changes in inflammatory mediators, differentiation markers and fibro-adipogenic progenitor (FAP)-associated genes [by reverse transcription quantitative PCR (RT-qPCR)].

Immunofluorescent staining of total macrophages (CD68) and anti-inflammatory M2 macrophages (CD206; also known as MRC1) was performed in muscle sections (Fig. 4A). In *Dysf^−/−^* and *Dysf^−/−^Dnm2^+/−^* sections, we observed an overall increase in both total and anti-inflammatory macrophages, with most CD68, but not CD206, staining detected next to dying cells. Expression patterns of genes encoding different cytokines were not significantly different between *Dysf^−/−^* and *Dysf^−/−^Dnm2^+/−^* samples, and revealed upregulation of *Tnfα* (also known as *Tnf*) *Il10* and *Tgfβ* (also known as *Tgfb1*) compared to those in WT and/or *Dnm2^+/−^* muscles (Fig. 4B), suggesting the co-existence of pro- and anti-inflammatory signals, with a potential predominance of anti-inflammatory responses.

**Fig. 4.**
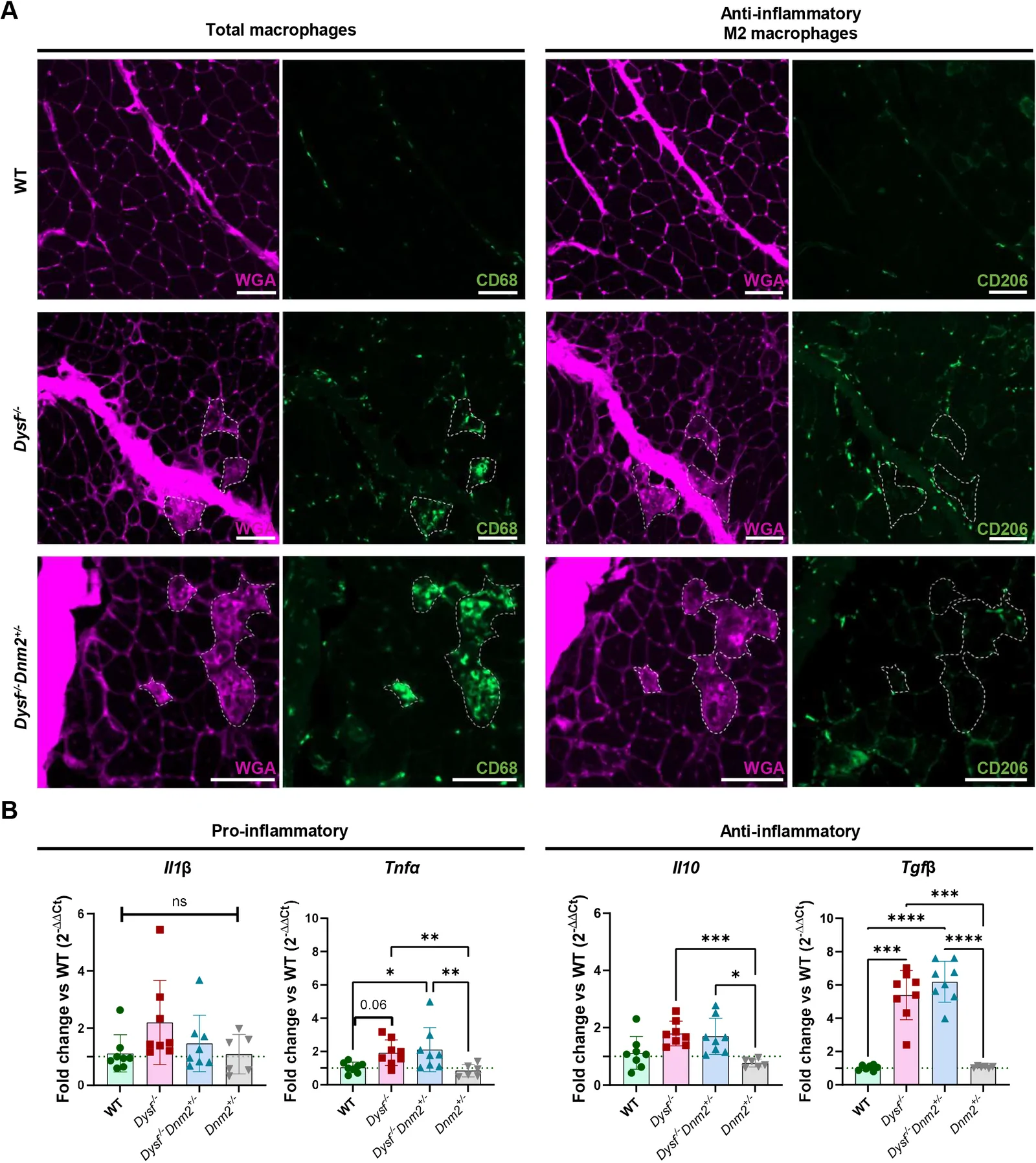
**DNM2 downregulation does not impact the inflammatory status of *Dysf^−/−^* mice.** (A) Representative images of immunofluorescent analysis of total macrophages (CD68) and anti-inflammatory macrophages (CD206) in 1-year-old quadriceps sections (representative of *n*=3 per genotype). White dashed lines outline dying cells. (B) Quantification of pro-inflammatory and anti-inflammatory cytokine mRNAs. Ordinary one-way ANOVA (*Il1b*, *Tnfα*) or Brown–Forsythe and Welch test (*Il10*, *Tgfβ*). *n*=7-8 (WT), 8 (KO; KOHET), 6 (HET). ns, not significant; *P<0.05; **P<0.01; ***P<0.001; ****P<0.0001. Each dot represents a mouse. KO, *Dysf^−/−^*; KOHET, *Dysf^−/−^Dnm2^+/−^*; HET, *Dnm2^+/−^*; WGA, wheat germ agglutinin. Reverse transcription quantitative PCR (RT-qPCR) analyses were replicated two to three times.

To repair or renew muscle fibers, satellite cells can be activated, transitioning from a quiescent stem-cell state marked by *Pax7* expression to a proliferative state marked by *MyoD* (also known as *Myod1*) and, ultimately, to differentiating myogenic cells marked by *Myog*. In our study, *Pax7* expression was increased in *Dysf^−/−^* mice compared with that in WT mice, but *MyoD* mRNA levels were not significantly different between the genotypes, suggesting a potential increase in satellite cell abundance without elevated activation (Fig. 5A). In contrast, *Myog* expression was increased 7-fold relative to that in WT, suggesting increased ongoing myogenic differentiation. *Pax7* and *Myog* expression in *Dysf^−/−^Dnm2^+/−^* was not significantly different from that in *Dysf^−/−^* mice, suggesting no strong effect of the treatment. The distinct expression pattern of *MyoD* and *Myog* could be consistent with altered myogenic maturation observed in dysferlin-deficient cultures (Vallecillo-Zúniga et al., 2020).

**Fig. 5.**
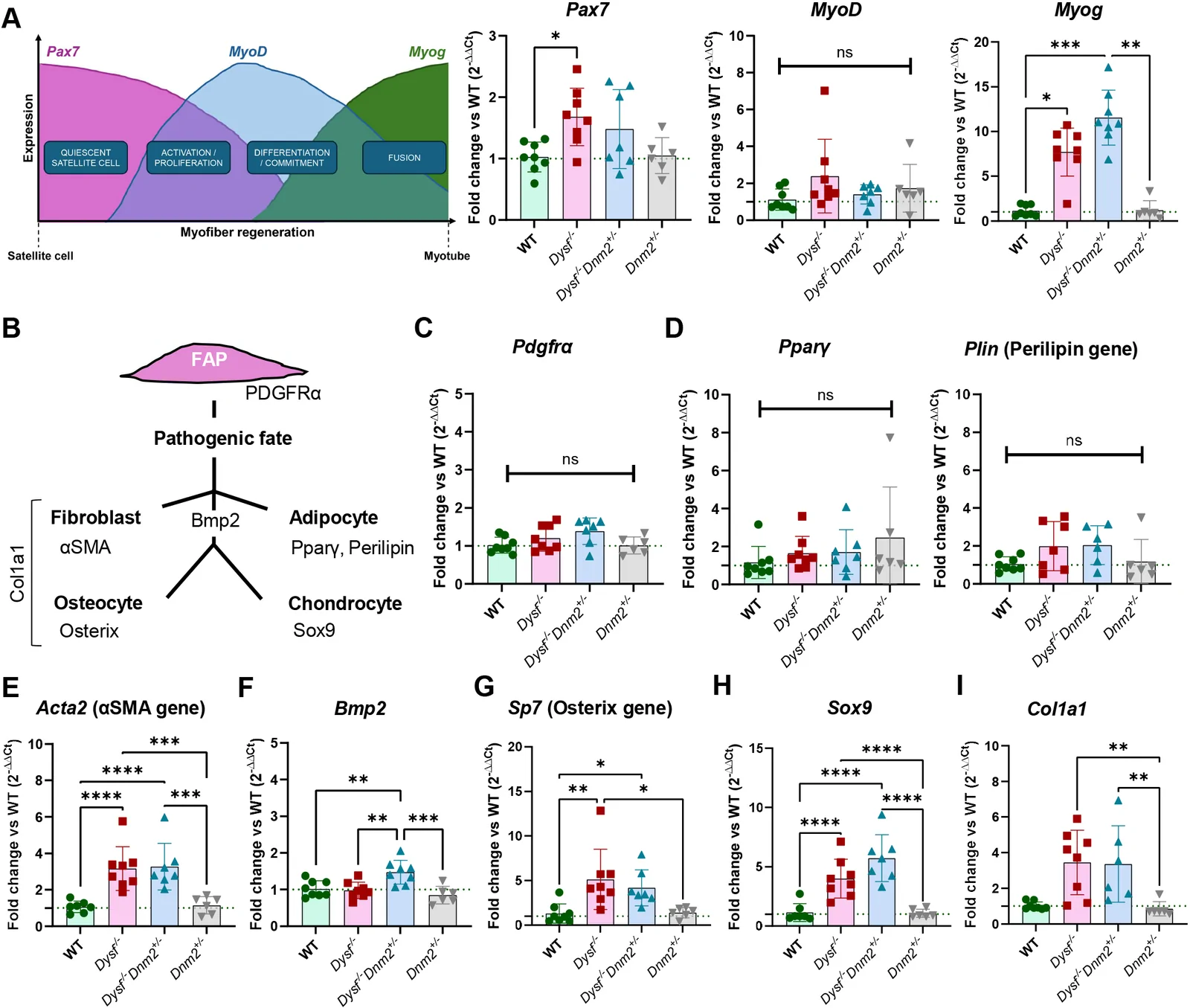
**DNM2 downregulation does not prevent abnormal gene expression associated with myogenic maturation and FAP fate.** (A-I) RNA was extracted from quadriceps muscles at 1 year of age. (A) Schematic representation of muscle fiber regeneration factors and quantification of associated gene expression levels. One-way ANOVA (*Pax7*, *MyoD*) or Kruskal–Wallis (*Myog*). *n*=WT; KO (8), KOHET (7-8), HET (6). (B) Schematic representation of FAP pathological fates and associated genes (Giuliani et al., 2022; Contreras et al., 2021). (C-I) Quantification of mRNAs associated with FAP fates. Kruskal–Wallis (*Sp7* and *Col1a1*) or one-way ANOVA (other mRNAs). *n*=7-8 (WT); 7-8 (KO), 6-8 (KOHET), 6 (HET). ns, not significant; *P<0.05; **P<0.01; ***P<0.001; ****P<0.0001. Each dot represents a mouse. FAP, fibro-adipogenic progenitor; KO, *Dysf^−/−^*; KOHET, *Dysf^−/−^Dnm2^+/−^*; HET, *Dnm2^+/−^.* RT-qPCR analyses were replicated two to three times.

In dystrophic conditions, FAPs proliferate, notably through anti-apoptotic signaling, and commit to pathological fates, including fibroblastic differentiation with collagen production, as well as adipocytic, osteocytic or chondrocytic differentiation (Hogarth et al., 2025; Lemos et al., 2015) (Fig. 5B). Here, we did not observe any significant difference in expression of the FAP marker *Pdgfra* between genotypes (Fig. 5C), or in the expression of genes associated with adipogenic fate [*Pparg* and *Plin* (also known as *Plin1*)] (Fig. 5D). However, we observed an increase in *Acta2* in *Dysf^−/−^* and *Dysf^−/−^Dnm2^+/−^* samples compared to that in WT or *Dnm2^+/−^* samples, suggesting an increase in fibroblastic fate, consistent with the increase in Sirius Red staining (Fig. 5E). *Bmp2*, promoting chondro-osteogenic differentiation, was upregulated in *Dysf^−/−^Dnm2^+/−^* samples only, and may explain the calcification observed with Alizarin Red staining (Fig. 5F). Although *Sp7* and *Sox9* expression were significantly increased in *Dysf^−/−^* and *Dysf^−/−^Dnm2^+/−^* samples (∼5-fold compared to that in WT), indicating increased osteogenic and chondrogenic signatures, respectively, no significant difference was observed between *Dysf^−/−^* and *Dysf^−/−^Dnm2^+/−^* samples (Fig. 5G,H). Finally, *Col1a1* was upregulated in *Dysf^−/−^* and *Dysf^−/−^Dnm2^+/−^* samples compared to that in *Dnm2^+/−^* samples, consistent with a potential increase in fibroblasts and osteocytes (Fig. 5I).

Overall, these results reinforce the dystrophic phenotype of both *Dysf^−/−^* and *Dysf^−/−^Dnm2^+/−^* muscles, characterized by inflammatory infiltration, myogenic differentiation and increased expression of genes linked to pathological FAP fates. Although the histological data strongly suggest a worsening of the phenotype upon DNM2 downregulation in dysferlin-deficient muscle, the gene expression profiles were broadly similar, except for *Bmp2*, which was specifically upregulated in *Dysf^−/−^Dnm2^+/−^* muscle compared to that in *Dysf^−/−^* and control muscles.

### Opposing genetic modulations of BIN1 and DNM2 produce similar molecular perturbations in *Dysf^−/−^* muscle

Downregulating DNM2 or overexpressing BIN1 similarly rescued the phenotypes of CNM mouse models. Here, both modulation strategies share common deleterious effects in the *Dysf^−/−^* mouse model of dysferlinopathy.

As with BIN1 overexpression, Cav-3 protein levels were significantly increased in *Dysf^−/−^Dnm2^+/−^* mice compared to those in WT and *Dnm2^+/−^* controls. Of note, *Cav3* mRNA expression was also increased in the *Dysf^−/−^Dnm2^+/−^* mice compared to that in mice of the other groups (Fig. 6A,B).

**Fig. 6.**
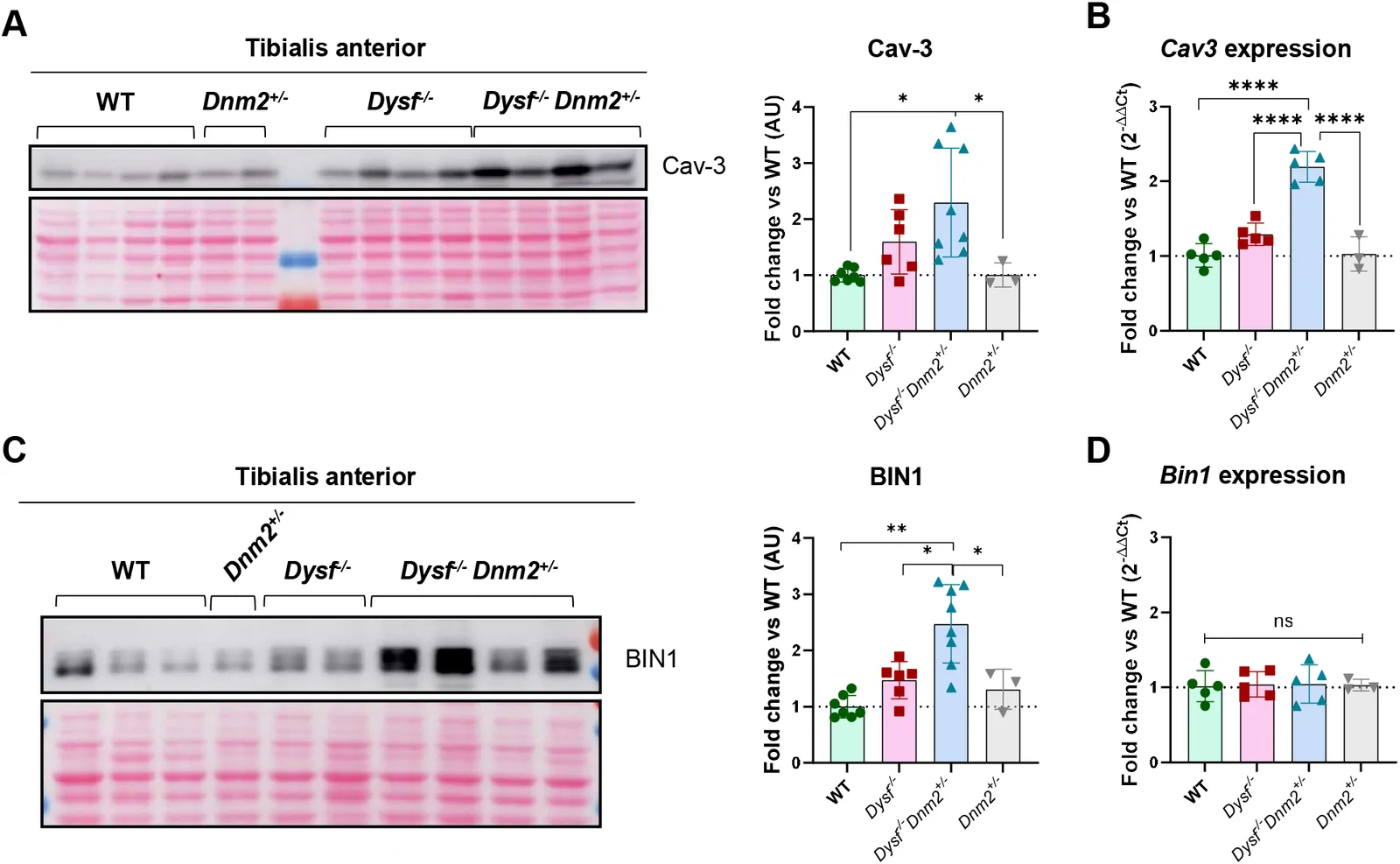
**DNM2 downregulation leads to dysregulations in BIN1 and Cav-3 protein levels.** (A-D) Quantification of BIN1 and Cav-3 protein (A,C) and mRNA (B,D) levels in the TA at 1 year of age, representative of three independent experiments. (C) BIN1 detection was performed on the same membrane as the one used in Fig. 3A. (A,C) Brown–Forsythe and Welch test. *n*=7 (WT); 6 (KO), 8 (KOHET), 3 (HET). (B,D) Ordinary one-way ANOVA. *n*=5 (WT; KO; KOHET), 3 (HET). ns, not significant; *P<0.05; **P<0.01; ****P<0.0001. Each dot represents a mouse.

In the *Dysf^−/−^TgBIN1* mice, DNM2 was significantly increased compared to that in *Dysf^−/−^* and TgBIN1 control mice. In the *Dysf^−/−^Dnm2^+/−^* mice, we found a 2.4-fold increase in BIN1 protein compared to that in WT, with no difference at the gene expression level (Fig. 6C,D).

In summary, both BIN1 and DNM2 modulation resulted in increased protein levels of BIN1, DNM2 and Cav-3, revealing a shared dysregulation of membrane remodeling proteins that may underlie phenotype exacerbation.

## DISCUSSION

### Increasing BIN1 or downregulating DNM2 have similar effects in *Dysf^−/−^* mice

In this study, we evaluated whether two therapeutic strategies successfully applied in CNMs, namely BIN1 overexpression and DNM2 downregulation, could ameliorate muscle defects in a dysferlin-deficient context. Contrary to our initial hypothesis, both approaches exacerbated functional and histopathological features in *Dysf^−/−^* mice. BIN1 overexpression failed to improve muscle force and increased circulating MYOM3 levels, and fibrosis. DNM2 downregulation led to an even more pronounced deterioration, including more severe muscle weakness, elevated MYOM3, increased necrosis and the emergence of muscle fiber calcification.

Importantly, both genetic modulations converged on a similar molecular signature characterized by increased protein levels of BIN1, DNM2 and Cav-3, suggesting shared dysregulation of membrane remodeling pathways in dysferlin-deficient muscle. Interestingly, DNM2 elevation was observed in untreated *Dysf^−/−^* mice and in biopsies from patients with dysferlinopathy, indicating that its upregulation precedes experimental intervention and is intrinsic to the disease context. Together, these findings challenge the assumption that strategies effective in T-tubule-related myopathies can be directly translated to dysferlinopathy and instead suggest a disease-specific role for DNM2 in maintaining muscle integrity under conditions of membrane instability.

### Context-dependent consequences of BIN1–DNM2 modulation in muscle disease

The BIN1–DNM2 axis plays a central role in membrane remodeling and T-tubule biogenesis in skeletal muscle, and its dysregulation is a well-established driver of CNMs. In CNMs, *DNM2* gain-of-function variants or increased DNM2 activity disrupt membrane trafficking and intracellular organization, leading to defective E-C coupling (Cowling et al., 2011). In this context, either reducing DNM2 or increasing BIN1, an endogenous inhibitor of DNM2, restores intracellular organization and improves muscle force in multiple genetic forms of the disease. These observations have positioned DNM2 normalization as a robust and broadly applicable therapeutic strategy for CNM (Zhao et al., 2018).

Our findings indicate that this therapeutic logic does not extend to dysferlinopathy. As in CNM, we observed elevated DNM2 protein levels in severely affected dysferlin-deficient muscle in mice, as well as in patient biopsies. However, in contrast to CNM, for which excessive DNM2 activity is causative, dysferlinopathy is primarily characterized by defective membrane repair and chronic sarcolemmal instability. These distinct pathogenic drivers likely determine the divergent consequences of modulating the BIN1–DNM2 axis.

Mechanistically, although both CNM and dysferlinopathy involve T-tubule abnormalities, the initiating events differ. In CNM, impaired membrane curvature and triad organization directly compromise E-C coupling. In dysferlinopathy, repeated membrane injury and insufficient repair impose sustained mechanical and metabolic stress on muscle fibers. In such a context, membrane remodeling proteins such as DNM2 may contribute to compensatory responses aimed at maintaining membrane integrity and adapting to chronic damage. Reducing DNM2 levels under these conditions may, therefore, impair adaptive remodeling processes rather than correct a primary pathogenic driver.

Taken together, these data underscore that the functional outcome of BIN1 or DNM2 modulation is highly disease-context dependent. The same molecular pathway that is pathogenic in one myopathy can be protective or compensatory in another, emphasizing the necessity for tailoring therapeutic strategies to the specific pathomechanism of each disorder.

### DNM2 increase is a potential compensatory mechanism in dysferlinopathy

Because BIN1 inhibits DNM2, both BIN1 overexpression and direct DNM2 downregulation are expected to reduce DNM2 functional activity. Given that DNM2 is a dose-sensitive protein, and *DNM2* gain-of-function variants cause CNM and loss-of-function variants lead to peripheral neuropathy, its reduction in dysferlin-deficient muscle may have deleterious consequences. Our data suggest that a relative *DNM2* loss of function may underlie the worsening observed with both modulation strategies.

Dysferlin deficiency impairs membrane repair and exposes muscle fibers to chronic sarcolemmal stress. In this context, sustained membrane remodeling is likely to be required to relieve tension and maintain integrity. DNM2, beyond its role in T-tubule biogenesis, regulates membrane fission, endocytosis and vesicle trafficking, processes that may become particularly important in the absence of efficient repair (Puri et al., 2020; Bitoun et al., 2009). The elevation of DNM2 in untreated *Dysf^−/−^* mice and in biopsies from patients with dysferlinopathy suggests that its upregulation is intrinsic to the disease and may represent an adaptive response.

Consistently, DNM2 downregulation led to fiber calcification, suggestive of intracellular calcium overload or increased fiber injury leading to niche dysregulation. This second hypothesis could be supported by the increase in *Bmp2* gene expression in *Dysf^−/−^Dnm2^+/−^* quadriceps, hinting at FAP differentiation toward chondro-osteocytic fate. However, other chondro-osteocytic markers were not significantly increased in *Dysf^−/−^Dnm2^+/−^* compared to *Dysf^−/−^* quadriceps. Additionally, niche maladaptation is unlikely to be the sole contributor to the phenotype exacerbation, as direct DNM2 reduction significantly decreased strength in the TA muscle of *Dysf^−/−^* mice, which retains most of its histological integrity compared to proximal muscles. Interestingly, although DNM2-dependent endocytosis does not appear to mediate the immediate resealing of membrane wounds, evidence supports a slower phase of post-repair remodeling involving DNM2 (McDade et al., 2021). DNM2 may, therefore, contribute to membrane stabilization and replenishment of intracellular membrane pools after injury, rather than to initial closure. In dysferlin-deficient muscle, reducing DNM2 could compromise this secondary remodeling phase, increasing *in fine* calcium influx, potentially leading to contractile defects and accelerating degeneration. Further studies are needed to investigate whether membrane permeability is increased with DNM2 reduction in dysferlin-deficient conditions.

Nonetheless, these findings support the notion that DNM2 upregulation in dysferlinopathy serves a compensatory function rather than a pathogenic one.

### Role of Cav-3: secondary response or pathogenic contributor?

Cav-3 protein levels were increased in *Dysf^−/−^* mice with both BIN1 overexpression and DNM2 downregulation, indicating that its upregulation accompanies the aggravated phenotypes. However, this elevation is unlikely to be specific to either modulation strategy, as Cav-3 is also increased in other muscular disorders, including BIN1-related CNM (Giraud et al., 2023) and Duchenne muscular dystrophy (Repetto et al., 1999; Waddell et al., 2011). This suggests that Cav-3 upregulation reflects global membrane stress rather than a primary pathogenic event.

In *Dysf^−/−^TgBIN1* mice, Cav-3 overexpression could, nonetheless, contribute to disease worsening, as excessive Cav-3 has been shown to impair myoblast fusion, another muscle repair mechanism, and promote fibrosis (Galbiati et al., 2000; Volonte et al., 2003). In contrast, following DNM2 downregulation, Cav-3 was increased at both protein and mRNA levels, consistent with a transcriptional response that may represent compensation to altered membrane dynamics. Overall, Cav-3 elevation likely reflects a secondary adaptation to membrane remodeling imbalance, although it may further aggravate pathology under certain conditions.

### Concluding remarks

BIN1 overexpression and DNM2 downregulation have proven effective in rescuing T-tubule and locomotor defects in CNMs. However, our study demonstrates that, in the context of dysferlin deficiency, these same strategies are deleterious to muscle force and structural integrity. These results indicate that therapies targeting the BIN1–DNM2 pathway may not work the same way in all muscle diseases, emphasizing that membrane remodeling mechanisms are highly dependent on the specific disease context. Importantly, the intrinsic upregulation of DNM2 in dysferlin-deficient muscle suggests that it may fulfill an adaptive role in maintaining membrane integrity under chronic injury. Notably, this study suggests that muscle weakness progresses with age in *Dysf^−/−^* mice, while the onset of functional impairment differs between males and females. Therefore, although neither strategy provided a functional benefit, these findings should be interpreted with caution. Together, our data support an intricate relationship between dysferlin and DNM2 that extends beyond T-tubule biogenesis and indicate that precise, context-specific modulation, rather than simple reduction, of DNM2 activity may be required for therapeutic benefit in dysferlinopathy.

## MATERIALS AND METHODS

### Mouse models

The dysferlin-deficient BLA/J mouse model, coming from the introgression of the *prmd* allele into the C57BL/6J background by the team of Isabelle Richard (Lostal et al., 2010), was supplied by The Jackson Laboratory. *TgBIN1* mice were created through the insertion of human bacterial artificial chromosome (no. RP11-437K23; Grch37 chromosome 2: 127761089-127941604) encompassing the full *BIN1* gene and were previously described (Lionello et al., 2019). The *Dnm2^+/−^* mice were created by CRE-LoxP deletion of *Dnm2* exon 8 in the Sv124Pas background (Cowling et al., 2014). Experiments were conducted on both males and females at 6 or 12 months of age. The target sample size was based on sample sizes used in previous studies (Nagy et al., 2017) and on the availability of animals generated through the breeding scheme. A maximum of eight animals per sex and genotype was used where sufficient animals were available. This study was conducted in accordance with the French legislation under the ethical committee agreement 41415-2023022216052223.

### Patient biopsies

Skeletal muscle biopsies were collected for diagnostic purposes, in accordance with French regulations, and obtained from the Unite de Morphologie Neuromusculaire at the Institut de Myologie. Open muscle biopsies were taken from the deltoid muscle and snap frozen in liquid nitrogen-cooled isopentane. All patients provided written informed consent for the reuse of their biological samples and clinical data for research purposes. In France, additional approval from an ethics committee is not required for the secondary research use of diagnostic samples with patient consent.

### *In situ* muscle force

TA force measurements were performed using a Complete 1300A Mouse Test System (Aurora Scientific). Mice were anesthetized with a combination of domitor/fentanyl (2/0.28 mg/kg) and diazepam (8 mg/kg), followed 15 min later by an additional injection of fentanyl (0.28 mg/kg) to achieve adequate narcosis, sedation, analgesia and muscle relaxation. Skin and muscle incisions were made at the hip level to expose the sciatic nerve. The distal tendon of the TA muscle was sectioned and attached to a force transducer using a nylon suture. The muscle was partially detached from surrounding tissues (approximately three-quarters of its length). The knee and paw were firmly immobilized throughout the experiment. The sciatic nerve was electrically stimulated at frequencies ranging from 2 to 150 Hz. Muscle-specific force was calculated by normalizing the absolute force to the mass of the TA muscle. The experimenter was unaware of the genotype.

### Tissue collection and histology

Dissected psoas, quadriceps and TA muscles were directly frozen in liquid-nitrogen cold isopentane. HE, Alizarin Red, Sirius Red and Oil Red O stainings were performed on 8 μm transversal cryosections cut with a Leica CM3050 S cryostat. Images were acquired an a Zeiss Axioscan 7, and the Cellpose algorithm was used to segment individual fibers. Nuclei internalization and necrosis quantifications were done on Fiji manually using CellCounter. Total and Alizarin Red-, Sirius Red- and Oil Red O-stained areas were measured in Fiji using Color Threshold and Analyze Particles functions. One transversal section was quantified for each animal. Sections with extensive freezing artifacts were excluded from quantification. Investigators were aware of genotypes during histological quantifications.

### Immunofluorescence

Longitudinal TA and transversal quadriceps cryosections (8 μm) were fixed in 4% paraformaldehyde for 20 min, permeabilized with 0.2% Triton X-100 (T8787, Merck) in PBS for 10 min, and blocked for 1 h in PBS containing 0.1% Triton X-100 and 5% bovine serum albumin (BSA; 5217, Tocris Bioscience). Sections were then incubated overnight at 4°C with an anti-DNM2, anti-CD68 or anti-CD206 primary antibody (Table S1), followed by incubation with an Alexa Fluor 488-conjugated secondary antibody (1:500; A11055, RRID#AB_2534102 or A11006, RRID#AB_2534074, Invitrogen) for 1 h 30 min at room temperature with Alexa Fluor 647-conjugated wheat germ agglutinin (WGA) (1:250; W32466, Invitrogen). Slides were mounted using Invitrogen™ ProLong™ Gold Antifade Mountant, and images were acquired with a Zeiss Observer Z1 microscope equipped with a 63× objective.

### RNA extraction and RT-qPCR

Frozen tissues were mechanically homogenized using a Precellys^®^ Evolution Touch system (Bertin Technologies) with two 15-s pulses at 5500 rpm in TRI Reagent (TR118, Molecular Research Center). Total RNA was extracted by chloroform phase separation, precipitated with isopropanol, washed with 75% ethanol and resuspended in DEPC-treated water. Complementary DNA (cDNA) was synthesized using SuperScript™ IV Reverse Transcriptase (18090010, Invitrogen). Quantitative real-time PCR was performed using SYBR Green Master Mix I (04887352001, Roche Diagnostics) on a LightCycler^®^ 480 system (Roche Diagnostics). All reactions were performed with both biological and technical replicates (two to three technical replicates per condition). Primers can be found in Table S1.

### Protein extraction and western blotting

Total proteins from tissue were extracted in RIPA buffer (150 mM NaCl, 50 mM Tris-HCl, pH 8.0, 0.5% sodium deoxycholate, 1% NP-40 and 0.1% SDS) supplemented with 1 mM PMSF, 1 mM sodium orthovanadate, 5 mM sodium fluoride, 0.2 mM DTT, and a 1× protease inhibitor cocktail. Tissue samples were mechanically homogenized using a Precellys^®^ Evolution Touch system (Bertin Technologies) with two 20-s pulses at 6000 rpm, and supernatant was kept after centrifugation at 10,000 ***g*** for 5 min. Protein concentrations were determined using a DC Protein Assay Kit (5000116, Bio-Rad). Proteins were reduced in Pierce™ Lane Marker Reducing Sample Buffer (39000, Thermo Fisher Scientific) and heated at 95°C for 5 min before loading on the gel. Proteins (10-15 μg per lane) were loaded on polyacrylamide gels prepared according to standard protocols and transferred onto nitrocellulose membranes using a Trans-Blot^®^ Turbo RTA Mini Nitrocellulose Transfer Kit (170-4270, Bio-Rad) for 10-12 min. For MYOM3, the same protocol was followed except for the fixed volume of plasma used (1 μl reduced in 8 μl of Pierce™ Lane Marker Reducing Sample Buffer with 31 μl of water). Membranes were stained with Ponceau S for total protein visualization. After blocking for 1 h in 5% nonfat dry milk or BSA in Tris-buffered saline containing 0.1% Tween-20 (TBST), membranes were incubated overnight at 4°C with primary antibodies diluted in 5% milk or BSA in TBST. Membranes were washed three times for 5 min in TBST and incubated for 1 h at room temperature with the appropriate horseradish peroxidase-conjugated secondary antibodies. Chemiluminescent signals were detected following a 2-min incubation with SuperSignal™ West Pico PLUS substrate (34580, Thermo Fisher Scientific), and images were acquired using an Amersham Imager 600 (GE Healthcare Life Sciences). Band intensities were quantified using Fiji software and normalized to total protein levels (Ponceau S staining) and to the mean value of the control group to calculate fold-change values. Primary antibodies are listed in Table S1. Uncropped images are available in Figs S7-S10.

### Statistical analysis

Statistical analyses and graph generation were performed using GraphPad Prism (version 10.0.2). Data normality was assessed using the Shapiro–Wilk test (*n*<8) or D'Agostino–Pearson test for datasets with *n*≥8. For normally distributed data with homogeneous variances, unpaired *t*-test (*n*=2) or one-way ANOVA (*n*>2) were applied. When variance homogeneity was not met, unpaired *t*-test with Welch's correction (*n*=2) or Brown–Forsythe and Welch ANOVA (*n*>2) were used. Log-normally distributed data were log transformed prior to statistical analyses. Non-normally distributed datasets were analyzed using Mann–Whitney (*n*=2) or Kruskal–Wallis (*n*>2) tests. For each ANOVA, multiple comparisons were conducted by comparing the mean of each group with the mean of every other group. Statistical significance was defined as *P*<0.05. Graphs display individual data points along with mean±s.d. All individual values and corresponding statistical methods are provided in Table S2. No method of randomization was implemented, as all animals and genotypes were generated through breeding crosses.

## Supplementary Material

10.1242/dmm.052918_sup1 Supplementary information

Table S2. Raw experimental data underlying all plotted graphs, together with the normality and statistical tests performed and the corresponding *P*-values for each comparison.
