# Supplementary material for: Increasing BIN1 or decreasing DNM2 worsens pathology in a mouse model of dysferlinopathy
Source: Dis Model Mech. 2026 Oct 26;19(10):dmm052918. doi: 10.1242/dmm.052918 (PMC13750400; doi:10.1242/dmm.052918)
Supplement: Supplementary file 1 [file dmm-19-052918-s1.pdf]

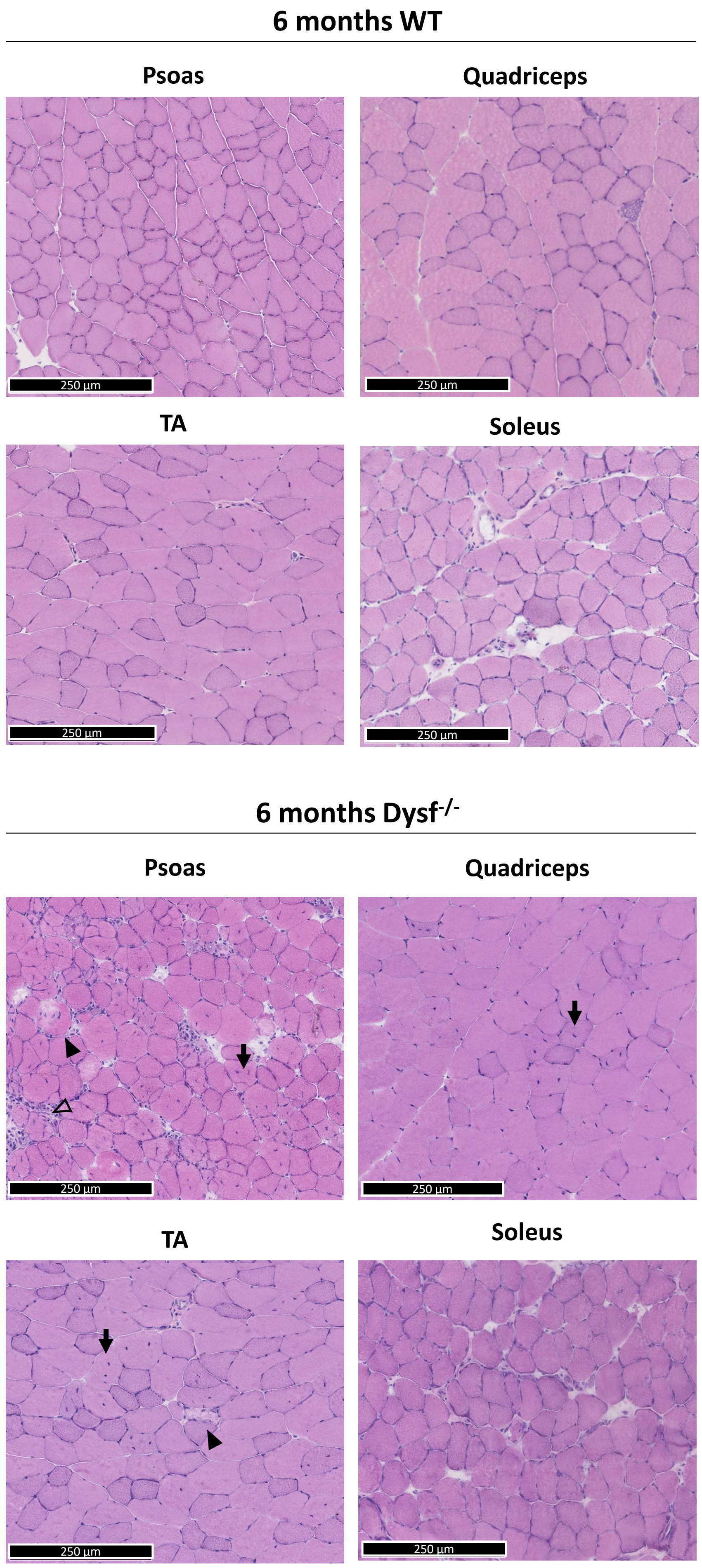

Fig. S1. Histopathology of different skeletal muscles in *Dysf$^{-/-}$* mice. Representative images of four different WT and *Dysf$^{-/-}$* skeletal muscles at 6 months of age. Black arrow indicates fibers with at least one internalized nuclei. Black arrowhead indicates necrosis. Empty arrowhead indicates immune cell infiltration. Scale bar = 250 nm.

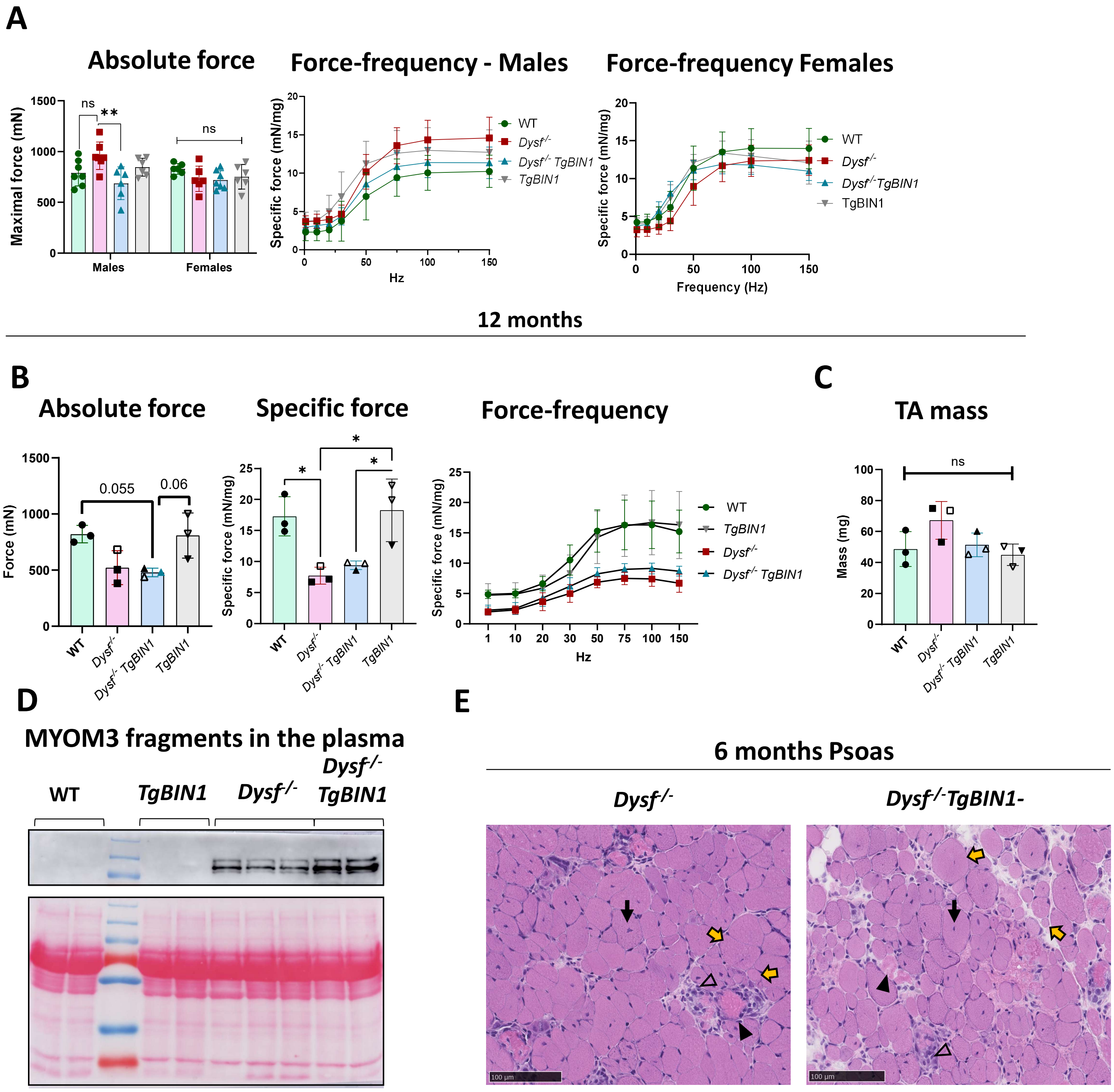

Fig. S2. BIN1 overexpression does not rescue muscle weakness and worsens histological phenotype. (A-B) *In situ* muscle force measurements in the tibialis anterior (TA) after sciatic nerve stimulation at 6 months (A) or 1 year (B). (A) n = WT;KO;KOTG (6- 7), TG (6). Ordinary one-way ANOVA with multiple comparison. (C) Mass of the TA used for *in situ* muscle force at 1 year. (B-C) Empty dots represent males in (B-C). n = 3. Ordinary one-way ANOVA with multiple comparison. (D) Representative image of MYOM3 fragments in the plasma quantified by western blot. (E) Representative image of transversal sections of the psoas muscles of *Dysf*$^{-/-}$ and *Dysf*$^{-/-}$*TgBIN1* mice. Black arrow indicates fibers with at least one internalized nuclei. Black arrowhead indicates necrosis. Empty arrowhead indicates immune cell infiltration. Yellow arrows indicate fiber diameter heterogeneity. Scale bar = 100 nm. . *p<0.05; **p<0.01. KO: *Dysf*$^{-/-}$; KOTG: *Dysf*$^{-/-}$*TgBIN1*; TG: *TgBIN1.*

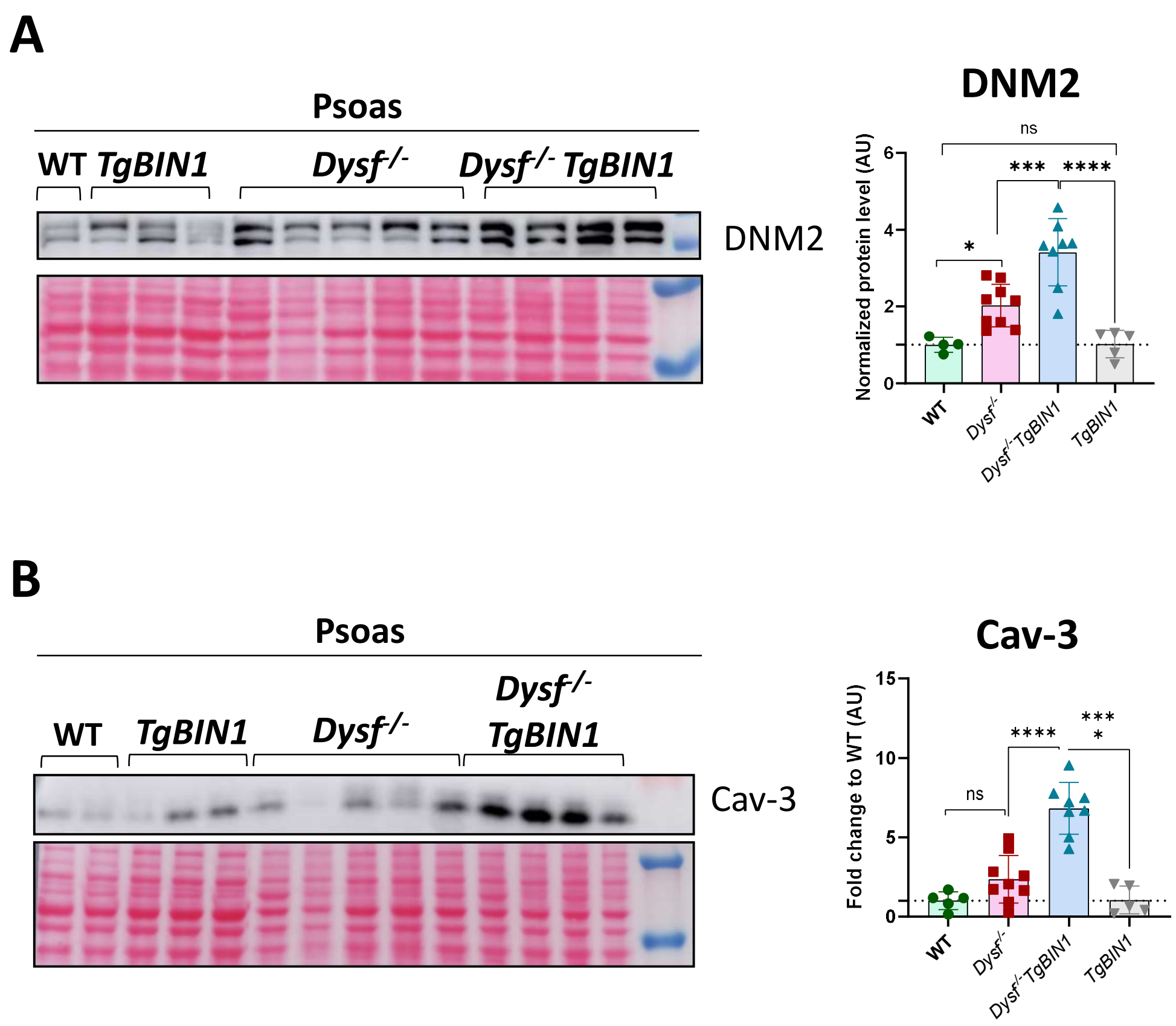

Fig. S3. DNM2 and Cav-3 protein levels in the psoas following BIN1 overexpression in *Dysf$^{-/-}$TgBIN1* mice. Quantification of DNM2 (A) and Cav-3 (B) protein levels in the psoas at 6 months of age. Ordinary one-way ANOVA. (A) n= WT (4), KO (9), KOTG (8), TG (5). (B) n= WT;TG (5), KO (9), KOTG (8). *p<0.05; ***p<0.001; ****p<0.0001. KO: *Dysf$^{-/-}$*; KOTG: *Dysf$^{-/-}$TgBIN1*; TG: *TgBIN1*.

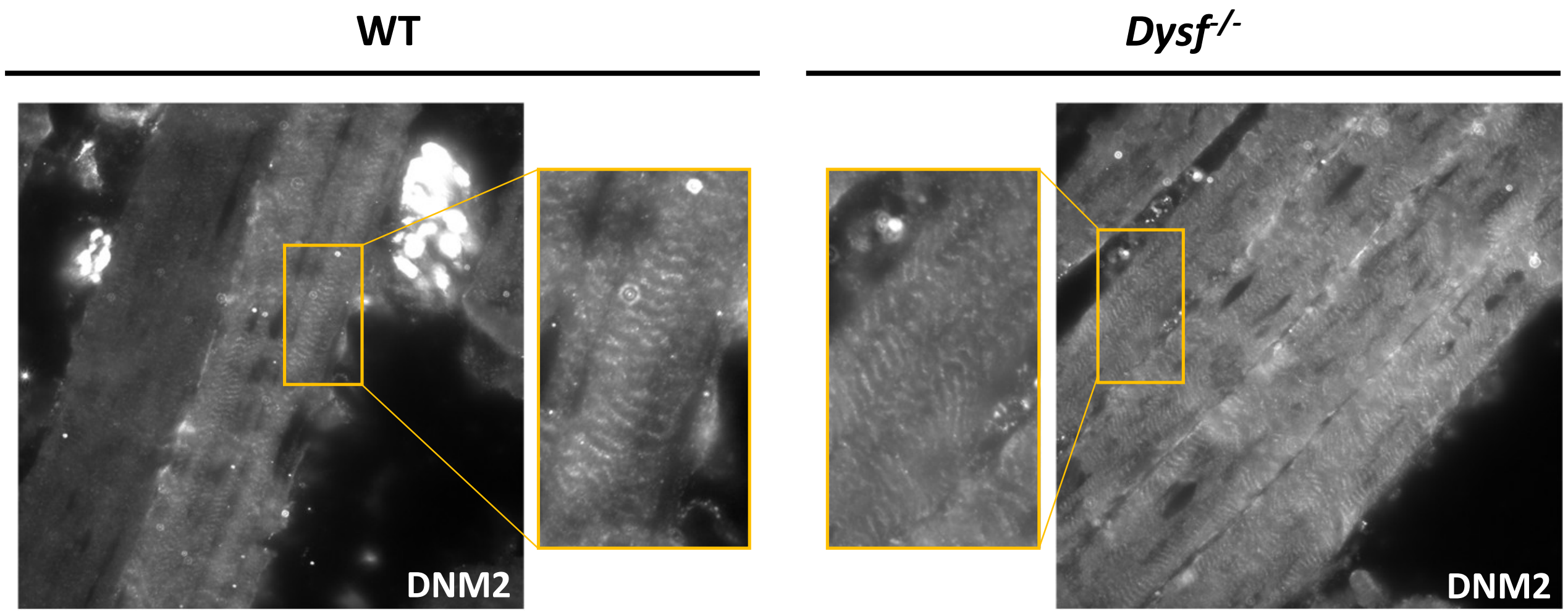

Fig. S4. DNM2 localization. Representative images of DNM2 immunostaining on longitudinal TA cryosection at 12 months of age (representative of n = 3 per genotype).

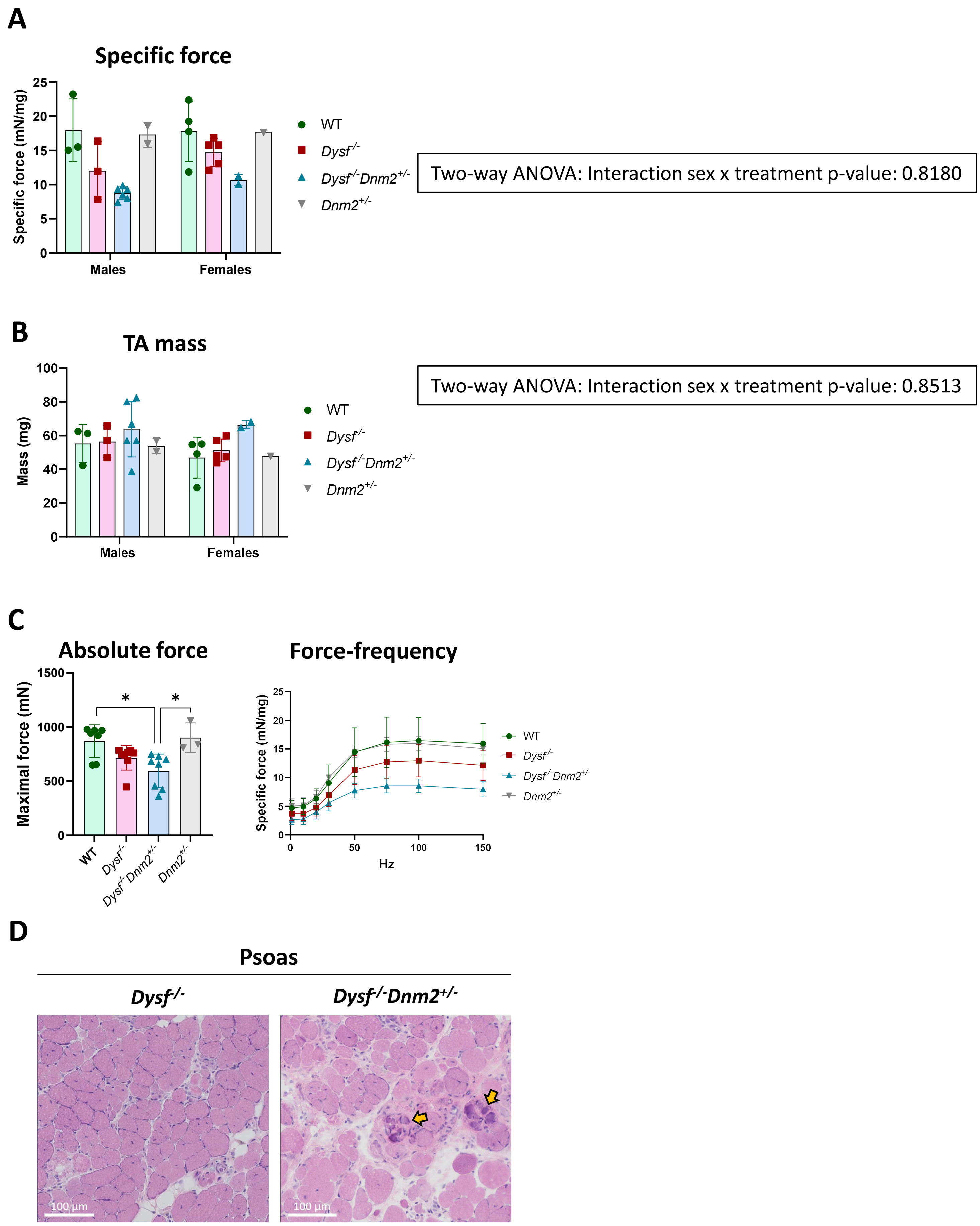

Fig. S5. DNM2 downregulation in the dysferlinopathie mouse. (A) *In situ* TA measurements at 1 year separated by sex (left) and effect of the sex on the TA force (right). (B) Mass of TA used for *in situ* specific force measurements. Each dot represents a mouse. (C) *In situ* TA measurements at 1 year: raw force (left) and specific force after sciatic nerve stimulation at increasing frequencies (right). n = WT (7), KO;KOHET (8), HET (3). Kruskal-Wallis with multiple comparison test. (D) Representative image of psoas transversal sections of one-year old *Dysf*$^{-/-}$ and *Dysf*$^{-/-}$*Dnm2*$^{+/-}$ mice. Yellow arrow indicates fiber calcification. *p<0.05. KO: *Dysf*$^{-/-}$; KOHET: *Dysf*$^{-/-}$*Dnm2*$^{+/-}$; HET: *Dnm2*$^{+/-}$.

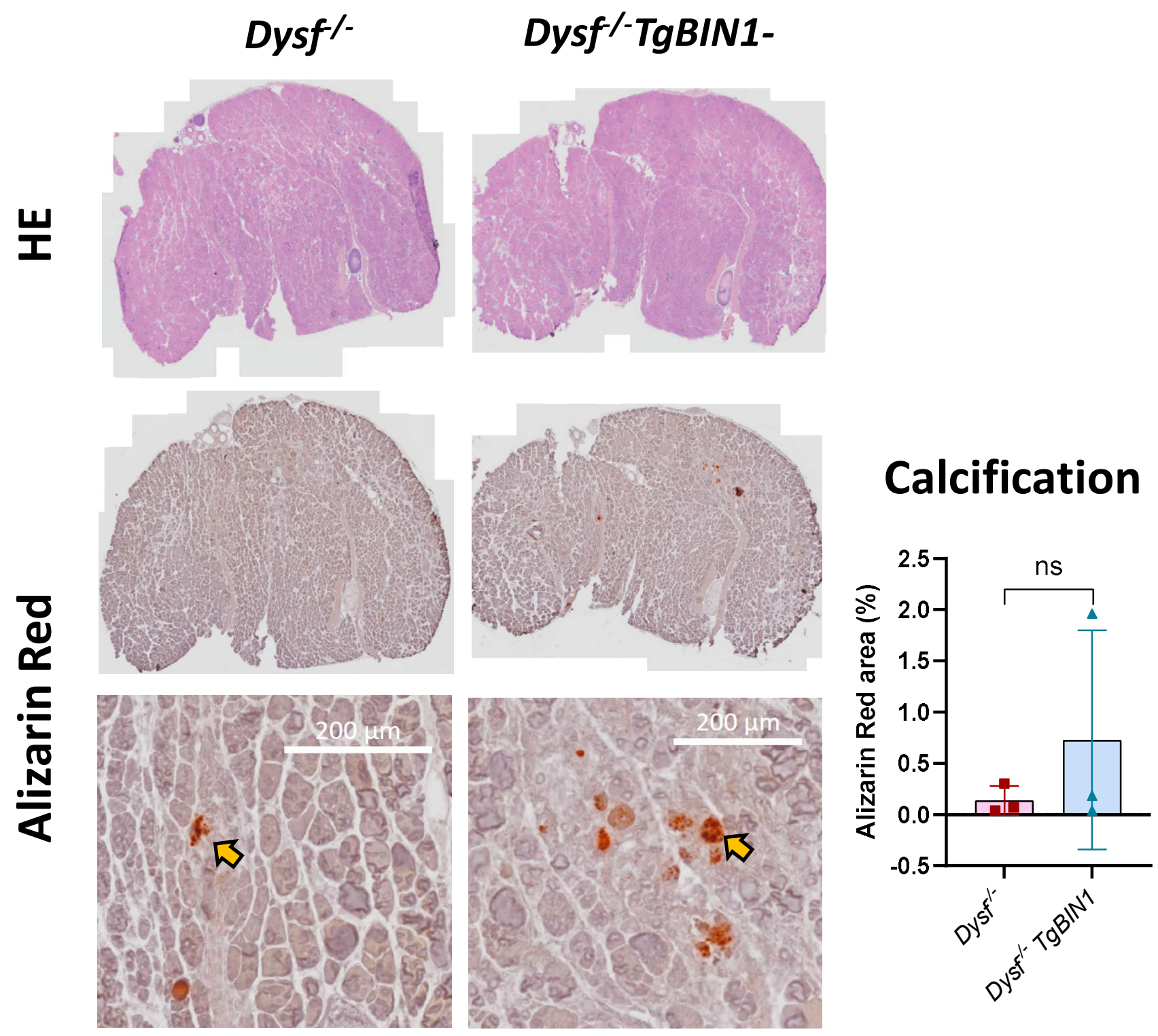

Fig. S6. BIN1 overexpression does not induce fiber calcification. Representative images of HE and Alizarin Red stained transversal quadriceps section at 1 year. Yellow arrow indicates fiber calcification. Fiber calcification is measured as the area occupied by Alizarin Red staining on total tissue area. n=3. Unpaired t-test with Welch's correction. Scale bar = 200 µm.

Disease Models & Mechanisms • Supplementary information

Table S1. Antibodies used for western blots or immunofluorescence, and primers used for qPCRs.

**Western blot antibodies**	
BIN1 – SH3 domain (1:1000)	Homemade (3623)
Caveolin-3 (1:1000)	Abcam (ab171752)
DNM2 (1:1000)	Homemade (2865)
MYOM3 (1:1000)	Euromedex (176921-AP)
**Immunofluorescence antibodies**	
DNM2 (1:150)	Santa Cruz Biotechnology (C18 sc-6400)
CD68 (1:200)	Bio-Rad (MCA1957GA)
CD206 (1:150)	BioLegend (141703)
**qPCR primers**	**5' → 3'**
Housekeeping gene	
Rps11 -FOR	CGC GTG GTG AAT AAG GAA GC
Rps11 -REV	GTA AGC ACG CTC CGT CTG AA
Genes of interest	
Bin1 (exon 9) - FOR	GTCAACACGTTCCAGAGCATCG
Bin1 (exon 10) - REV	CCTTGACTGTGAAGGTGTTGCTC
Dnm2 (exon 6) - FOR	ACCCCACACTTGCAGAAAAC
Dnm2 (exon 8) - REV	CGCTTCTCAAAGTCCACTCC
Cav-3 (exon 1) - FOR	GATAGACTTGGTGAACCGCGAC
Cav-3 (exon 2) – REV	ACTTGGAGACGGTGAACGTGGT
Il1β - FOR	GCAACTGTTCCTGAACTCAACT
Il1β - REV	ATCTTTTGGGGTCCGTCAACT
Tnfα- FOR	GTTCTATGGCCCAGACCCTCAC
Tnfα- REV	CCCAGGTATATGGGCTCATACC
Il10 - FOR	GTCATCGATTTCTCCCCTG
Il10 - REV	CACCTTGGTCTTGGAGCT
Tgfβ – FOR	GATACGCCTGAGTGGCTGTC
Tgfβ - REV	CACAAGAGCAGTGAGCGCTG
Pax7 - FOR	GTTCGGGAAGAAAGAGGACGAC
Pax7- REV	GGTTCTGATTCCACATCTGAGCC
MyoD- FOR	CATCCGCTACATCGAAGGTC
MyoD- REV	CGCTGTAATCCATCATGCC
MyoG - FOR	CCCAACCCAGGAGATCATTTG
MyoG - REV	GGCAACAGACATATCCTCC
Pdgfrα – FOR	GTGAAGATGCTCAAACCCAC
Pdgfrα- REV	CTCCCAGCAAGTTCACAATG
Col1a1 - FOR	GTTCAGCTTTGTGGACCT
Col1a1- REV	CAGCTGACTTCAGGGATG
Acta2- FOR	CCATCTTTCATTGGGATGG
Acta2- REV	GGACGTTGTTAGCATAGAG
Pparγ - FOR	GACCAGGGAGTTCCTCAAA
Pparγ - REV	CAGGTTGTCTTGGATGTCCTC
Plin – FOR	GCTGTCTGAGACTGAGGTG
Plin- REV	GCCCTTGTTCATTGACATCC
Bmp2- FOR	CCATCACGAAGAAGCCGT
Bmp2- REV	GCAGATGTGAGAAACTCGTC
Sp7 - FOR	AAGGGTGGGTAGTCATTTGCA
Sp7 - REV	CCCTTCTCAAGCACCAATGG
Sox9 – FOR	CACCCCGATTACAAGTACCA
Sox9 - REV	CTGAGATTGCCCAGAGTGC

Table S2. Raw experimental data underlying all plotted graphs, together with the normality and statistical tests performed and the corresponding *P*-values for each comparison.

Available for download at
https://journals.biologists.com/dmm/article-lookup/doi/10.1242/dmm.052918#supplementary-data

Disease Models & Mechanisms • Supplementary information

Fig. S7. WB membranes – Fig 1

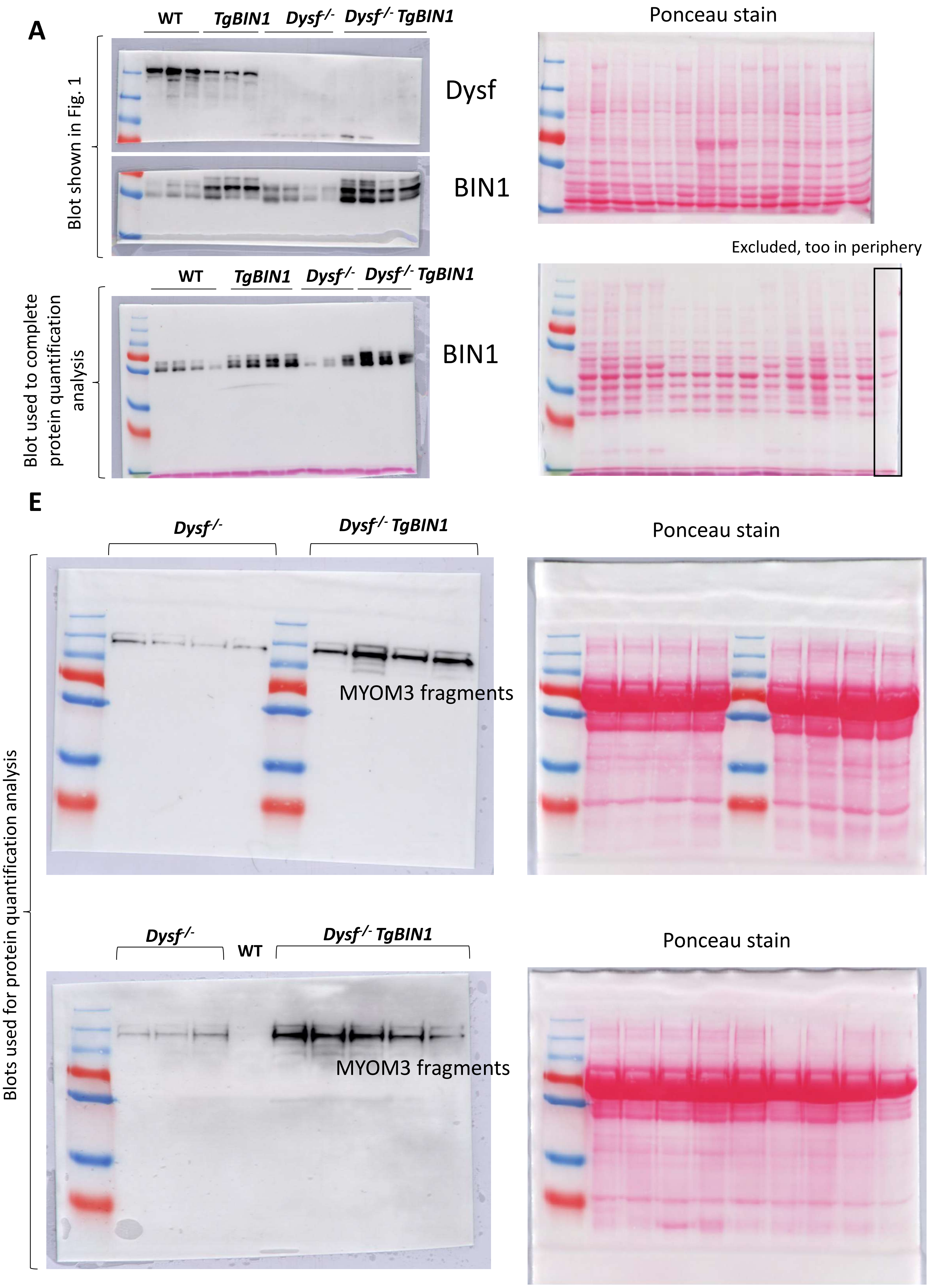

Disease Models & Mechanisms • Supplementary information

Fig. S8. WB membranes – Fig 2

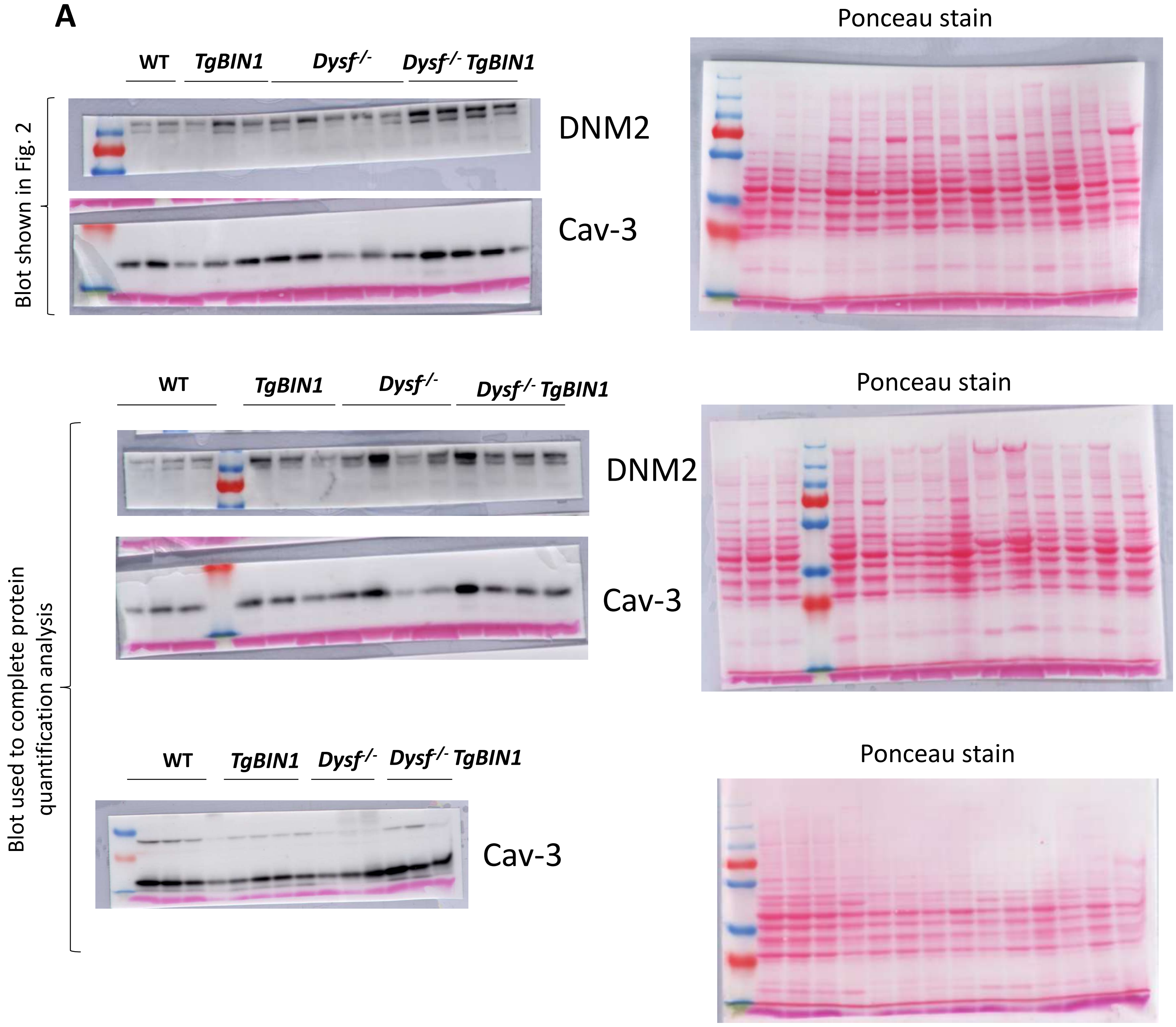

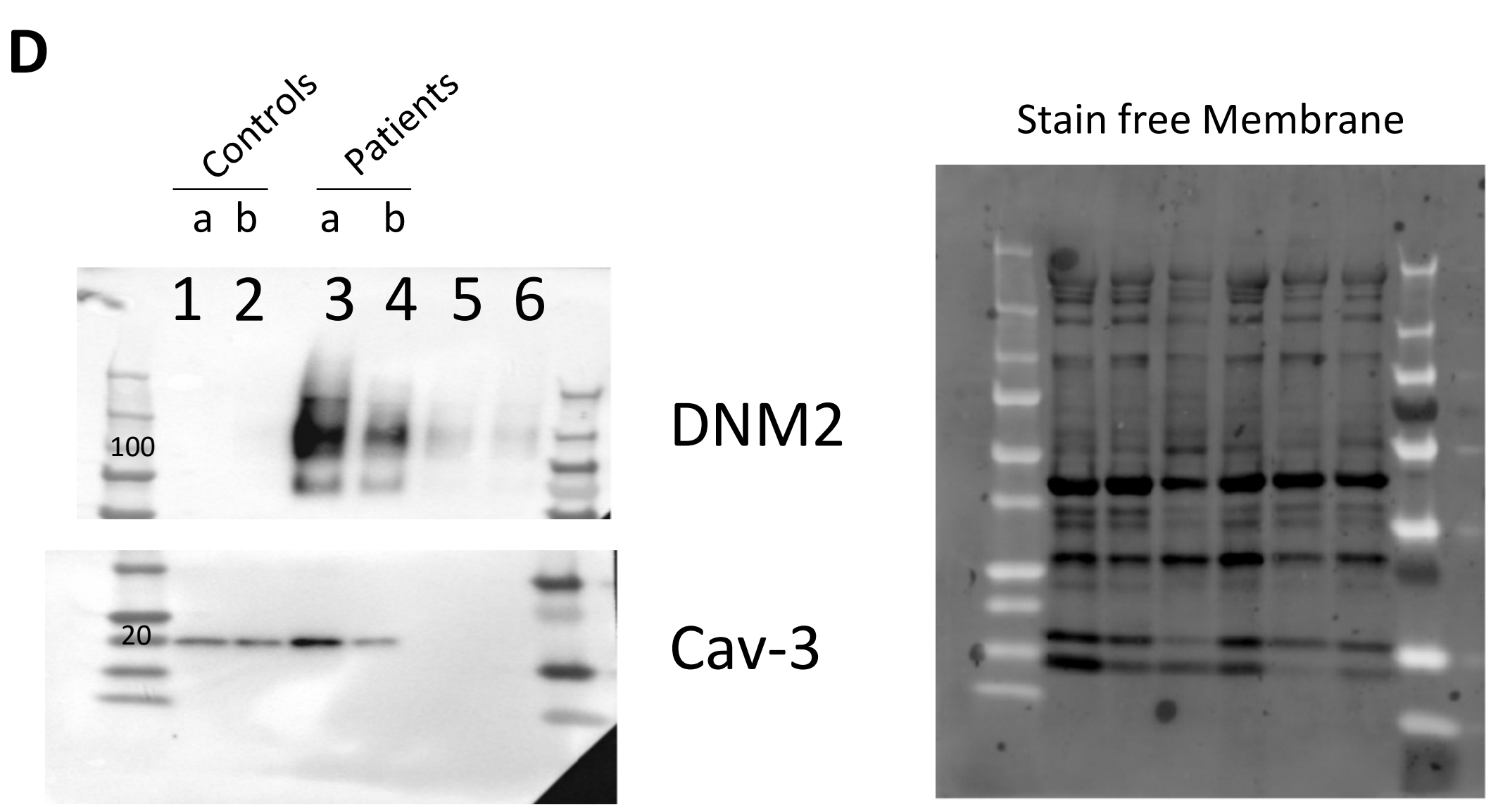

Fig. S9. WB membranes – Fig 3

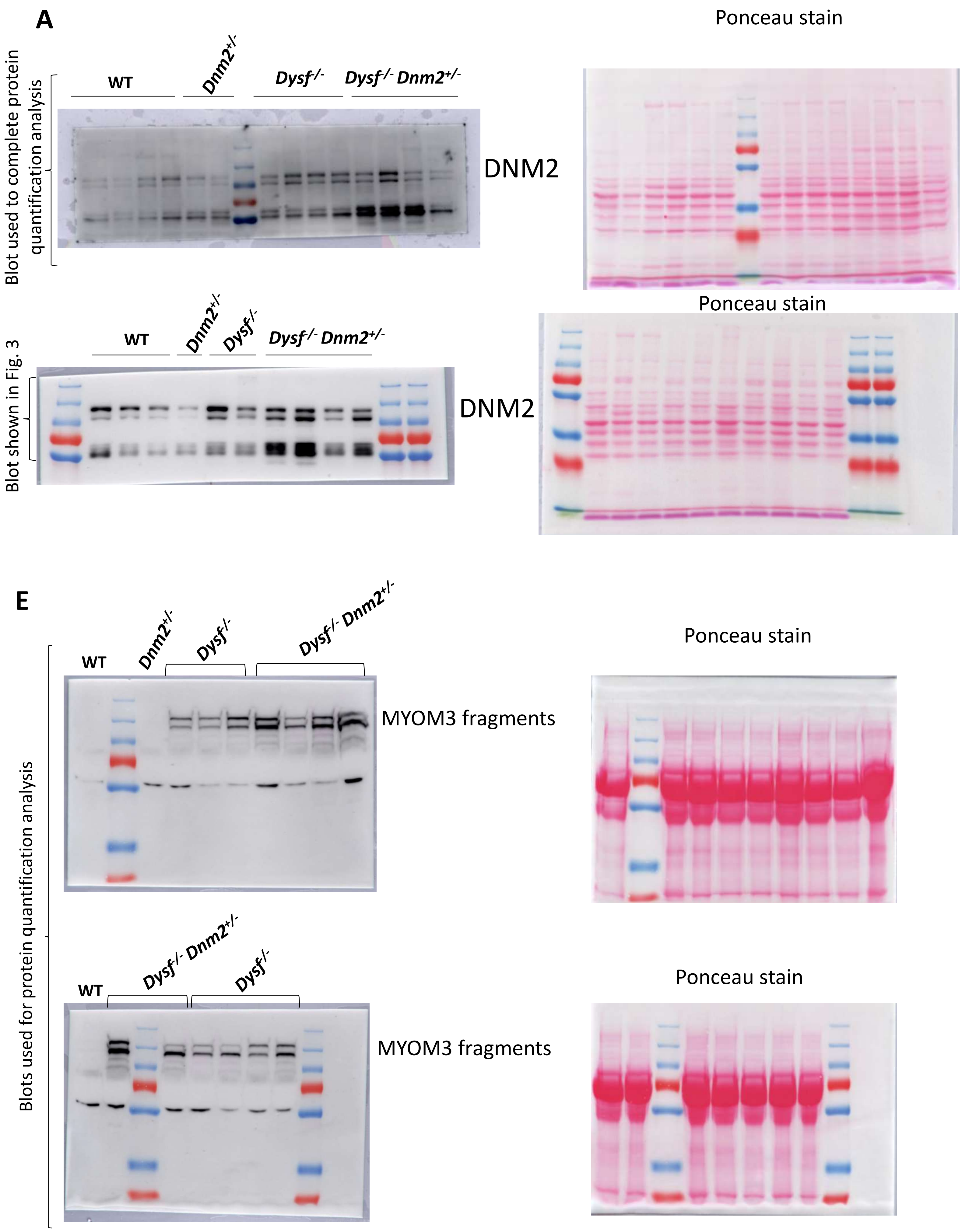

Fig. S10. WB membranes – Fig 6

A

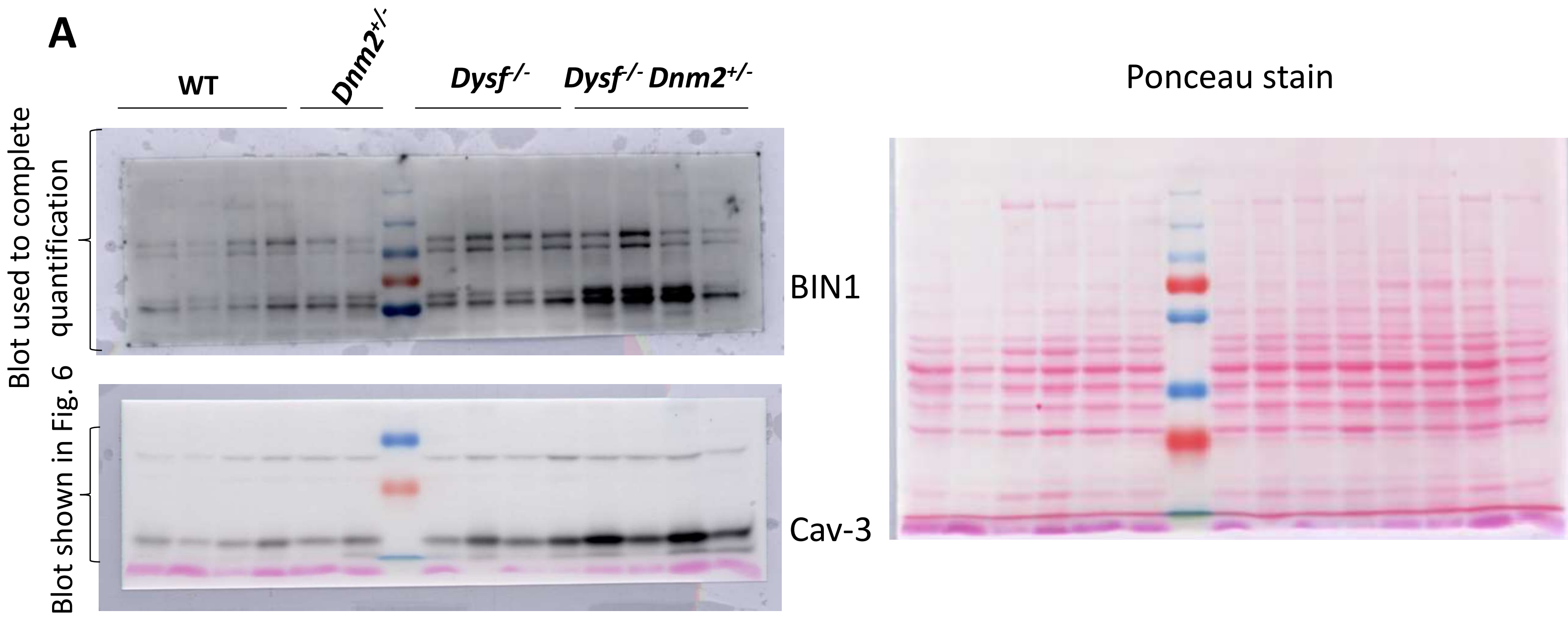

C

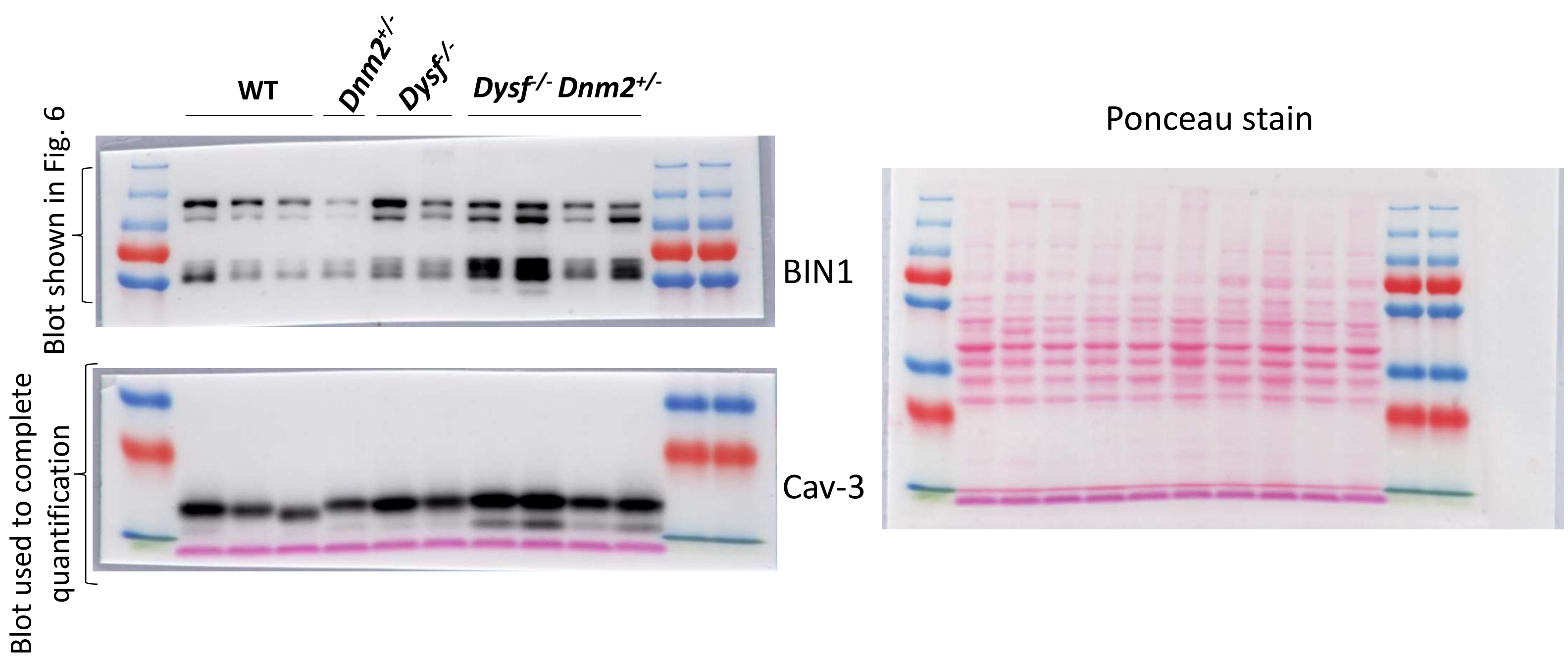

Disease Models & Mechanisms • Supplementary information
